# Supplementary material for: Evolution of new regulatory functions on biophysically realistic fitness landscapes
Source: Nat Commun. 2017 Aug 9;8:216. doi: 10.1038/s41467-017-00238-8 (PMC5548793; doi:10.1038/s41467-017-00238-8)
Supplement: Supplementary file 1 — Supplementary Information [file 41467_2017_238_MOESM1_ESM.pdf]

File name: Supplementary Information
Description: Supplementary figures, supplementary table, supplementary notes, supplementary methods and supplementary references.

File name: Peer review file
Description:

Evolution of new regulatory functions on biophysically realistic fitness landscapes
Supplementary Information

Tamar Friedlander, Roshan Prizak, Nicholas H. Barton and Gašper Tkačik

Supplementary Note 1 Model description and parameters

Biophysical model

Consider a transcription factor (TF) that activates n_G (≥ 2) downstream genes. The starting point of our evolutionary model is a duplication event of the TF, where the duplicate is fixed in the population. Gene regulation is accomplished by the binding of either TF (original or duplicate) to a short DNA sequence of length L associated with the gene (abbreviated below as 'BS': binding site). For simplicity we assume each gene has only a single BS. We describe the DNA-binding preference of each TF by its (unique) consensus sequence - the L-base-pair sequence to which it binds with highest affinity. We begin by assuming that each TF has only a unique consensus sequence and later on relax this assumption (see Supplementary Note 6). In our simple model, a gene is activated when its BS is bound by an activating TF. The probability that the binding site of gene j is bound by either TF is calculated using the thermodynamic model of gene regulation [1, 2]:

$$p_{jm}(\{k_{ij}\}, \{C_i(m)\}) = \frac{\sum_i C_i(m)e^{-\epsilon k_{ij}}}{1 + \sum_i C_i(m)e^{-\epsilon k_{ij}}}, \quad \text{(S1)}$$

where $\{k_{ij}\}_{i=1}^{2}$ is the number of sequence mismatches between the consensus sequence of the i-th TF species and the binding site of the j-th gene and ϵ is the energy per mismatch. We consider multiple environments m that differ in TF concentrations: $C_i(m)$ is the dimensionless concentration of the i-th TF in environment m. Associated with each TF i is an associated (complex) allele σ_i that determines the TF concentration $C_i(m)$ in different environments. Eq. (S1) assumes that all base pairs have equal and additive contributions to the binding energy, such that the binding probability only depends on the number of mismatches k_{ij} [2, 3, 4, 5].

Together, the TF consensus sequences, the BS sequences and the complex alleles σ_i compose the genotype. Genotypes come from the space of all possible genotypes $\mathcal{D}$, and they completely describe the regulatory activity of the system in different environments.

We study two variants of the model, depending on whether σ_i is evolvable or not.

Main model

In this model variant, which is described in the main text, transcription factors are equipped with an evolvable signal sensing domain (captured by σ_i). The original TF senses two distinct external signals. Each of the downstream genes is suitable to respond to only one of the two signals. Before duplication the genes are constrained to follow the only TF available which responds to both signals. The extra TF formed in the duplication event offers an additional degree of freedom in regulating these genes, if the TFs specialize such that each of them senses only one of the two signals and regulates only a subset of the genes.

This model variant is applicable to more general pathway architecture than a TF that implements both signal sensing and gene regulation in the same molecule. Often these two functions are split between different components of the same pathway; for example, a separate upstream component senses the signal(s) and consequently activates the TF (e.g. by phosphorylation or another modification). Additionally, TF production is also regulated. One can also think of the evolution of the regulatory sequences of the gene coding for the TF in terms of our model. Since our model is defined in very general terms, it can capture such situations as well.

Alternative model

In the alternative model, which we explore in the SI, transcription factors have no explicit evolvable signal sensing domain (no complex allele σ_i associated with them), but can be expressed at different time or location as determined by $C_i(m)$. Before duplication the genes are constrained to follow the only TF available, and are thus expressed at the same time or location. After TF duplication, the two copies immediately specialize to be active at different time slots (different parts of the cell cycle, different phases of developmental process) or space (different tissues), and as such enable distinct expression patterns for the downstream genes. This variant is a limiting case of the main model, with the main difference being the lack of an evolvable TF signal sensing domain. It also acts as an approximation when the signal sensing domain evolves very quickly, resulting in a quick divergence of TF expression patterns.

Gene birth can occur via different biological mechanisms, some of them allowing for the emergence of slightly modified copies of original genes or allowing for different regulation of the same coding sequence. One such mechanism is called 'retroposition': creation of duplicate gene copies in new genomic positions through the reverse transcription of mRNAs from source genes (also known as RNA-based duplication or retroduplication) [6]. These newly formed genes often lack regulatory elements of the parental gene and may also be slightly modified due to transcription errors (that are significantly more common than DNA-duplication errors). It was shown that transcription of these so-called 'retrogenes' is very common and often relies on regulatory elements of neighboring genes [7].

Evolutionary model

We define fitness such that the specialized genotypes have higher fitness compared to the initial non-specialized genotypes. The fitness of a genotype equals the squared deviation of the actual expression p_{jm} from the ideal one p^*_{jm}, summed over all genes j and averaged over all environments m:

$$F = -s \sum_j \sum_m \alpha_m \beta_{jm} (p_{jm} - p^*_{jm})^2, \tag{S2}$$

where s denotes the selection intensity and α_m is the frequency of the m-th environment. We define environments by the presence or absence of the signals, which result in different active TF concentrations depending on their signal responsiveness. β_{jm} is the penalty for each type of deviation from the ideal expression level, allowing for diverse penalties for different genes or at different environments. For example, a gene which is not expressed when needed can incur a higher penalty than the expression of a gene that is not necessary in a given environment. To

capture these latter interactions, which we call crosstalk interactions, we exploited β_{jm} to tune the fitness penalty in Supplementary Note 3. Expression levels p_{jm} for a genotype are calculated using Eq. (S1) by obtaining the dimensionless concentrations of the TFs, $C_i(m)$, from their signal sensing alleles σ_i, and the mismatches, k_{ij}, from the TF consensus sequences and the BS sequences.

Note that the fixation probability in Eq. (S3) below, depends, via the fitness, and in turn via the binding probabilities, directly on the TFs' signal sensing alleles σ_i, and the mismatches k_{ij} of the BS sequences with the TF consensus sequences, but not on M, the match between the TF consensus sequences. But, as shown in Fig. 2 of the main text, the set of possible k_{ij}'s is constrained by M, and hence, there is implicit selection on M. Also, importantly, selection does not directly depend on the TFs and BSs, but only via their biophysical interaction to result in appropriate gene regulation, thereby requiring concerted evolution of TFs and BSs.

The evolutionary process proceeds via three types of mutations: The BS of each downstream gene can acquire point-mutations at rate μ; the consensus sequence of each TF can have point-mutations at rate $r_{\text{TF}}\mu$. These two mutation types can modify the (mis)match values M and k_{ij}. A third type of mutation exists in the first model variant: the signal-sensing domain of each TF has two components, each of them can alternate between two alleles (sensitive/ non-sensitive to one of the two signals) at rate $r_s\mu$. Owing to the faster time-scales over which gene regulation evolves, we consider only these types of mutations on the BSs and TFs. In particular, we assume no change in the coding regions of the downstream genes themselves, only in their regulation.

Putting the pieces together

In our main model, we consider $n_G = 2$ downstream genes (models considering larger sets of downstream genes are explored in Supplementary Note 5), each of which is equipped with a binding site of length L, and two signals, with the presence/absence of the first (second) signal requiring the expression/silencing of the first (second) gene. In other words, information should be passed from the first signal to the first gene and from the second signal to the second gene.

The presence ('1') or absence ('0') of these two signals defines the different environments $m \in \{00, 01, 10, 11\}$ that are possible, with α_m denoting the frequency of environment m. These probabilities can be expressed in terms of three important parameters - f_1, f_2, the frequencies of each signal, and ρ, the correlation between the signals. We have

$$
\begin{aligned}
\alpha_{11} &= f_1 f_2 + \rho\delta \\
\alpha_{10} &= f_1(1-f_2) - \rho\delta \\
\alpha_{01} &= f_2(1-f_1) - \rho\delta \\
\alpha_{00} &= (1-f_1)(1-f_2) + \rho\delta
\end{aligned}
$$

where $\delta = \sqrt{f_1 f_2 (1-f_1)(1-f_2)}$. The frequency of each signal can be obtained as $f_1 = \alpha_{10} + \alpha_{11}$ and $f_2 = \alpha_{01} + \alpha_{11}$. In the main text, we assume that both signals are present at equal frequencies, and that each signal is present (or absent) half of the time $f_1 = f_2 = 0.5$. Hence, we have

$$
\begin{aligned}
\alpha_{00} = \alpha_{11} &= \frac{1}{4}(1+\rho) \\
\alpha_{10} = \alpha_{01} &= \frac{1}{4}(1-\rho)
\end{aligned}
$$

Thus when the signals are uncorrelated ($\rho = 0$), we have $\alpha_{00} = \alpha_{10} = \alpha_{01} = \alpha_{11} = 1/4$. When

the signals are fully correlated ($\rho = 1$) we obtain $\alpha_{00} = \alpha_{11} = 0.5$ and $\alpha_{10} = \alpha_{01} = 0$ and vice versa for anti-correlation ($\rho = -1$). We explore asymmetric environments in Supplementary Note 2.

The information transmission between signals and genes is mediated by TFs which contain a signal-sensing domain and a DNA-binding domain. TFs, on sensing a signal, become active and can induce the expression of a gene by binding to its binding site. We define each TF i by its consensus sequence, the sequence of length L for which the TF has the highest affinity, and its signal sensing allele $\sigma_i \in \{00, 01, 10, 11\}$, which describes its responsiveness to the two signals. If a TF i is responsive to a signal and that signal is present in environment m, then its active dimensionless concentration is $C_i(m) = C_0$, and $C_i(m) = 0$ otherwise. For simplicity, we assume only these two concentration levels.

The regulatory network is described by its genotype, $\mathcal{D}$, consisting of the consensus sequences and the signal sensing alleles of the two TFs, and the BS sequences of the (two) genes. As described in Eq. (S1) and Eq. (1) of the main text, the probability p_{jm} that the binding site of gene j is bound in environment m depends on, apart from ϵ, the mismatches k_{ij} (which can be obtained from the genotype sequences) between the consensus sequence of TF i and the BS of gene j, and the signal sensing alleles σ_i which determine the active concentrations $C_i(m)$.

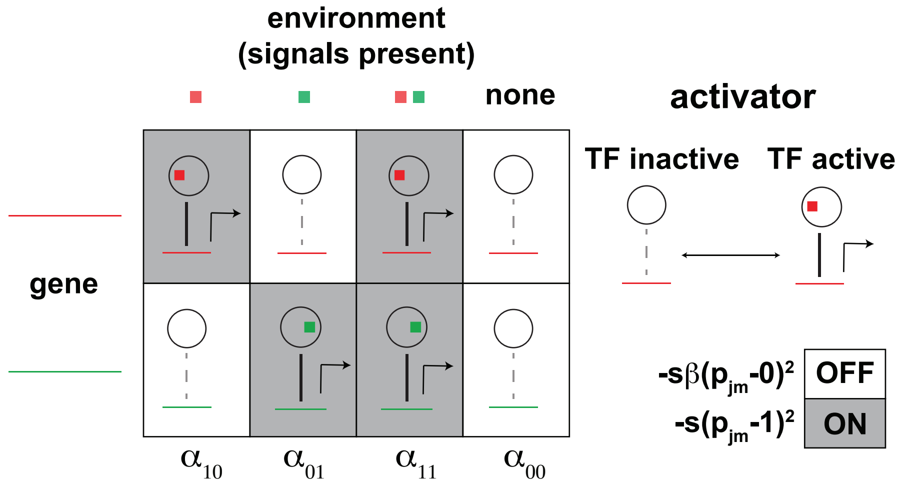

Supplementary Figure 1: **Optimal expression patterns and fitness contributions in different environments.** In the basic model, we consider only TFs that are activators. The downstream genes are by default inactive, unless activator TFs, triggered by input signals, bind to BSs and activate gene expression. Shown are the optimal expression patterns of the two genes in the four different environments, and the mechanistic aspects of the genotype that achieve these optimal patterns. Grey/white background - the gene is active/inactive; solid/dashed line - the TF binds/ does not bind the BS.

In Eq. (S2) and Eq. (2) of the main text, we define the fitness of a genotype by considering the deviation of the actual expression levels p_{jm} from the ideal expression levels p^*_{jm}. As shown in Suppl. Fig. 1, we define the ideal expression level of gene j in environment m, p^*_{jm}, such that $p^*_{jm} = 1$ if signal j is present in environment m and $p^*_{jm} = 0$ if signal j is absent in environment m. We consider the penalty $\beta_{jm} = 1$ if gene j is required in environment m and $\beta_{jm} = \beta_X$ ($\beta_X \in [0, 1]$) if gene j is not required in environment m. β_X quantifies the relative penalty on crosstalk interactions between signals and genes, compared to functional interactions. We explore the role of β_X in Supplementary Note 3. In Supplementary Table 1 we list the model parameters and their

baseline values used in calculations (unless stated otherwise).

Parameter	Explanation	Baseline value
L	BS length, length of sequences that TFs bind	5
ϵ	Energy contribution per bp towards TF-BS binding	$3\ k_BT$
C_0	Active TF free concentration in the presence of signal, set such that $p_{jm} = 0.5$ at $k = 1.5$	4.5
f_1	Frequency of signal 1	0.5
f_2	Frequency of signal 2	0.5
ρ	Correlation between input signals	0
β_X	β_{jm} when $p^*_{jm} = 0$ for gene j in environment m: penalty in fitness on activating a gene when it is not needed (crosstalk interaction)	0.5
other β	β_{jm} when $p^*_{jm} = 1$ for gene j in environment m: penalty in fitness on not activating a gene when it is needed (functional interaction)	1
Ns	Selection strength	25
r_S	Relative mutation rate of the signal sensing domain compared to the binding site mutation rate per bp	1
r_{TF}	Relative mutation rate of the TF consensus sequence per bp compared to the binding site mutation rate per bp	1

Supplementary Table 1: Model parameters and their baseline values.

With the fitness of genotypes and the mutations between them defined, we consider an evolutionary framework to study the evolutionary dynamics of this regulatory system. We assume mutation rates to be low enough such that a beneficial mutation fixes before an additional mutation (beneficial or not) arises. The condition under which this assumption is valid was found by Desai and Fisher [8] and reads $\frac{\log(4N\Delta F)}{\Delta F} \ll \frac{1}{4N\mu_b\Delta F}$. ΔF is the fitness advantage of the beneficial mutant, N is the population size and μ_b is the rate of beneficial mutations.

Under this condition the population is almost always fixed (monomorphic), and its evolutionary trajectory is captured by a series of discrete transitions between different genotypes. Consequently, when a new mutation emerges, it competes with only one other genotype. The fixation probability of a new mutation that alters the genotype from y to x equals

$$\Phi_{y\to x} = \frac{1 - \exp(-(F(x) - F(y)))}{1 - \exp(-2N(F(x) - F(y)))}, \tag{S3}$$

where the fitness F is defined by Eq. (S2) given the frequencies of the various environments α_m and the desired expression pattern of the genes p^*_{jm} at each. Eq. (S3) applies to a diploid population in which the mutant x appears in a single copy over a uniform background of the other genotype y. For diploids, the fitness difference $\Delta F = F(x) - F(y)$ refers to the fitness difference between the two homozygotes or to twice the selective advantage of the heterozygote (one copy of the mutant) over the prevailing homozygote genotype [9]. The overall rate of substitution from genotype y to x is given by [4]:

$$r_{xy} = 2N\mu_{xy}\Phi_{y\to x}, \tag{S4}$$

where μ_{xy} denotes the mutation rate from genotype y to x. We illustrate the evolutionary model further in Supplementary Note 7.

Space of reduced-genotypes

The size of the genotype space is huge, $|\mathcal{D}| = 4^{4L+2} \approx 10^{13.25}$ for $L = 5$, which makes it hard to analytically track the evolutionary model. Since the fitnesses of genotypes depend only on the mismatches k_{ij} and the signal sensing alleles σ_i, and the mutations only alter k_{ij}, σ_i and the TF consensus sequences' match M, we consider the space of "reduced-genotypes", $\mathcal{G} = \{M, k_{ij}, \sigma_i\}$, keeping track of only these reduced features of the genotype. The size of the reduced-genotype space is $|\mathcal{G}| < 16(L+1)^5 \approx 10^{5.09}$ for $L = 5$, which is tractable. Hence, for analytical calculations, we treat the regulatory network in the reduced-genotype space $\mathcal{G}$, and for simulations, we treat the regulatory network in the full genotypic space $\mathcal{D}$. Note that the reduced genotype representation in our model framework is *not* an approximation, but is an exact solution of the full genotype model, with the tractability gained due to clever bookkeeping of states in the sequence space.

Classification of genotypes into "macrostates"

Since our interest is in the biological function implemented by the network, we further coarse-grain the space of reduced-genotypes $\mathcal{G}$, and classify these reduced-genotypes into six possible macrostates, $\mathcal{M} = \{$`No Regulation`, `Initial`, `One TF Lost`, `Specialize Both`, `Specialize Binding`, `Partial`$\}$, by distinguishing only between "strong" and "weak" interactions. We set a threshold k_T and consider an interaction as weak, $k_{ij} \in \mathcal{W}$, if $k_{ij} > k_T$, and strong, $k_{ij} \in \mathcal{S}$, if $k_{ij} \leq k_T$. In the basic version of the model where both TFs have same biophysical properties (in particular same L) k_T is the same for all TF-BS interactions (but see the extension in Supplementary Note 6). The threshold k_T for each TF-BS pair ij is set such that for mismatches $k < k_T$, $p_{jm_i} \geq 0.5$ and for $k > k_T$, $p_{jm_i} < 0.5$ when only TF i is present and other TF(s) are absent, $C_i(m_i) = C_0$.

The full genotypic space $\mathcal{D}$ is a union of sequences belonging to different macrostates z:

$$\mathcal{D} = \bigcup_{z \in \mathcal{M}} S_z, \tag{S5}$$

where S_z is the set of all genotypes that belong to macrostate z. We apply the following classification rules.

`No Regulation`

The `No Regulation` macrostate consists of all genotypes in which there is no regulation of any form (no information transmitted from the signals to genes). This can happen if both the TFs either do not sense any signal or do not bind well to any binding sites.

$$x \in S_{\texttt{No Regulation}} \text{ if } \forall i \left((\forall j\ k_{ij} \in \mathcal{W}) \text{ OR } (\sigma_i = 00) \right) \tag{S6}$$

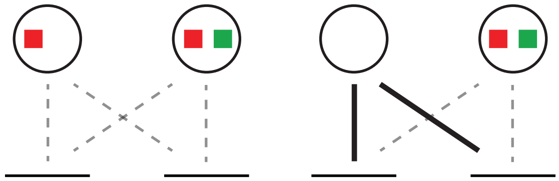

Supplementary Figure 2: **Typical genotypes in `No Regulation` macrostate.** In the left genotype, even though both TFs sense some signals, they do not bind well to either of the binding sites, hence preventing any information transmission. In the right genotype one TF binds both the binding sites but does not sense any signal and the second TF does not bind any binding site even though it senses both signals. This way or the other no information is transmitted between the signals and the genes.

`Initial`

The `Initial` macrostate consists of all genotypes in which there is complete regulation with no form of specificity: both the TFs sense both signals and bind both binding sites. This is the typical initial state right after duplication.

$$x \in S_{\texttt{Initial}} \text{ if } \forall i \left((\forall j \; k_{ij} \in \mathcal{S}) \text{ AND } (\sigma_i = 11) \right) \tag{S7}$$

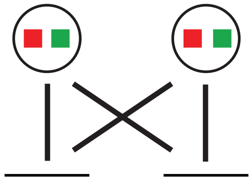

Supplementary Figure 3: **`Initial` macrostate genotypes.** In these genotypes, both TFs sense both signals and bind both binding sites.

`One TF Lost`

The `One TF Lost` macrostate consists of all genotypes in which one of the TFs is not involved in any regulation while the other is involved in some regulatory activity (namely, one TF does not sense any signal or does not bind well to any of the binding sites). This is equivalent to the genotypes before duplication, except that there is a "lost TF".

$$x \in S_{\texttt{One TF Lost}} \text{ if } \left| i : \left((\forall j \; k_{ij} \in \mathcal{W}) \text{ OR } (\sigma_i = 00) \right) \right| = 1 \tag{S8}$$

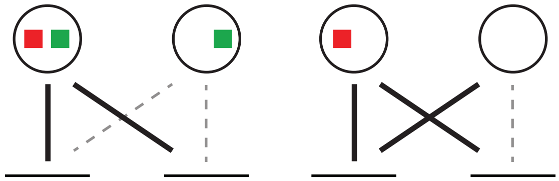

Supplementary Figure 4: **Typical genotypes in `One TF Lost` macrostate.** In the left genotype, only the first TF is involved in regulation as it senses both signals and binds to both binding sites. The second TF senses the green signal but does not bind any of the binding sites, hence it is not involved in regulation and is "lost". In the right genotype, again only the first TF is involved in regulation as it senses the red signal and binds both binding sites. The second TF not involved in any regulation because it does not sense any signal, although it binds the first binding site.

`Specialize Both`

The `Specialize Both` macrostate consists of all genotypes in which there is correct specialization of TFs with respect to both signal sensing and binding sites specificity. In these genotypes, one TF senses only the first signal and binds only to the first binding site, while the other TF senses only the second signal and binds only to the second binding site.

$$
\begin{aligned}
& x \in S_{\texttt{Specialize Both}} \text{ if} \\
& (k_{11}, k_{22} \in \mathcal{S} \text{ AND } k_{12}, k_{21} \in \mathcal{W} \text{ AND } \sigma_1 = 10 \text{ AND } \sigma_2 = 01) \\
\text{OR } & (k_{12}, k_{21} \in \mathcal{S} \text{ AND } k_{11}, k_{22} \in \mathcal{W} \text{ AND } \sigma_1 = 01 \text{ AND } \sigma_2 = 10)
\end{aligned} \tag{S9}
$$

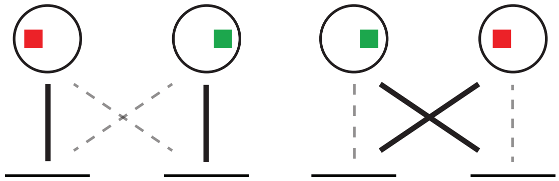

Supplementary Figure 5: **Genotypes in `Specialize Both` macrostate.** Both genotypes have specific paths from the signals to the genes. In the left genotype, while the first TF senses the red signal and binds the first (correct) binding site, the second TF senses the green signal and binds the second (correct) binding site. Hence, the first TF mediates the red signal to first gene pathway while the second TF mediates the green signal to second gene pathway. In the right genotype, the TFs exchange roles. The first TF mediates the green signal to second gene pathway while the second TF mediates the red signal to first gene pathway.

`Specialize Binding`

In contrast, the `Specialize Binding` macrostate consists of all genotypes in which there is specialization of TFs with respect to binding site specificities, but not with respect to the signal sensing domains.

$$x \in S_{\texttt{Specialize Binding}} \text{ if } (\forall i\ \sigma_i \neq 00) \text{ AND}$$
$$\Bigg(\Big((k_{11}, k_{22} \in \mathcal{S} \text{ AND } k_{12}, k_{21} \in \mathcal{W}) \text{ AND } \neg(\sigma_1 = 10 \text{ AND } \sigma_2 = 01)\Big)$$
$$\text{OR } \Big((k_{12}, k_{21} \in \mathcal{S} \text{ AND } k_{11}, k_{22} \in \mathcal{W}) \text{ AND } \neg(\sigma_1 = 01 \text{ AND } \sigma_2 = 10)\Big)\Bigg) \quad \text{(S10)}$$

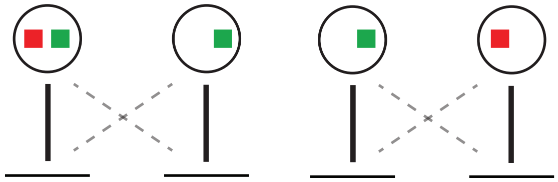

Supplementary Figure 6: **Typical genotypes in `Specialize Binding` macrostate.** In both genotypes, the first TF binds the first binding site and the second TF binds the second binding site, but they have not correctly specialized in their signal sensing domains. In the left genotype, while the second TF has specialized correctly to sense only the green signal, the first TF still senses both the signals. Hence, while the red signal pathway is established properly, the green signal pathway is not - both genes are activated in the presence of green signal. In the right genotype, the TFs have specialized in signal sensitivities, but opposite to the desired response pattern.

`Partial`

The `Partial` macrostate consists of all genotypes which do not belong in any of the other macrostates mentioned above. It contains a mixture of different regulatory architectures: both TFs regulate only one gene with the other gene unregulated, one TF regulates both genes while the other TF regulates only one gene or both TFs bind both binding sites but at least one TF has specialized in signal sensing.

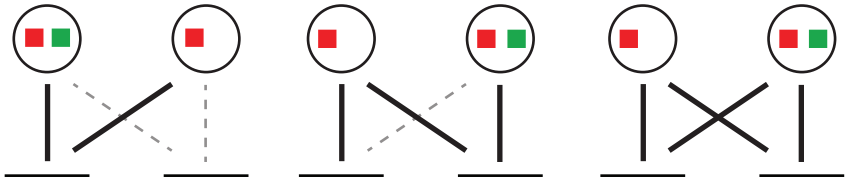

Supplementary Figure 7: **Typical genotypes in `Partial` macrostate.** In the left genotype, both TFs regulate only the first gene while the second gene is unregulated. In the middle genotype, the first TF regulates both genes while the second TF regulates only the second gene. In the right genotype, both TFs regulate both genes but, unlike the `Initial` macrostate, here the first TF does not mediate any information from the green signal.

Role of L in macrostate classification

Keeping ϵ and C_0 constant while changing L keeps the threshold mismatch k_T constant. Hence, the number of mismatches $|\mathcal{S}|$ in the strong binding class remains the same while the number of

mismatches $|\mathcal{W}|$ in the weak binding class increases. Hence, as L increases, the number of genotypes in all macrostates except `Initial` increase. The volume of macrostates with a larger number of weak mismatches increases more than the volume of macrostates with a smaller number of weak mismatches. For instance, `No Regulation` increases more than `Specialize Binding`. As `One TF Lost` and `Specialize Binding` have the same number of weak mismatches, the ratio of the number of genotypes in them stays the same for different L.

Supplementary Note 2 Steady state

The steady state distribution (Eq. (3) in the main text) is a general result in Population Genetics, derived as a solution of the forward Kolmogorov Equation [9]. It is a product of two factors: the neutral distribution (entropy term) and the fitness weight of different genotypes (energy term). The first factor, $P_0(\mathcal{G})$, is the neutral distribution (see below) which results from neutral processes only, such as mutation rates between different genotypes, assuming that all genotypes have equal fitness values. If fitness values are unequal, the second factor, $\exp(2NF(\mathcal{G}))$, biases the probabilities of attaining different genotypes accordingly. For a more comprehensive discussion and relation to statistical physics see Ref. [10].

Distribution of M for neutral and adaptive cases

In Fig. 3 of the main text we compared the steady state distribution of M (match between the two TFs) in the neutral case to the distribution of M if selection to diverge applies. Parameters used were $L = 5$, $Ns = 25$, resulting in hardly distinguishable distributions. Here we repeat this calculation with different parameter values that emphasize the difference between these cases: a stronger selection $Ns = 500$ and a longer binding site $L = 8$. A stronger selection depletes the highest match values compared to the neutral (Bernoulli) distribution. Even under these more extreme values the difference between the two distributions is modest, as shown in Suppl. Fig. 8. As a consequence, using distributions of M as estimated from genomic data may provide insufficient statistical power to detect selection pressure on TFs to diverge.

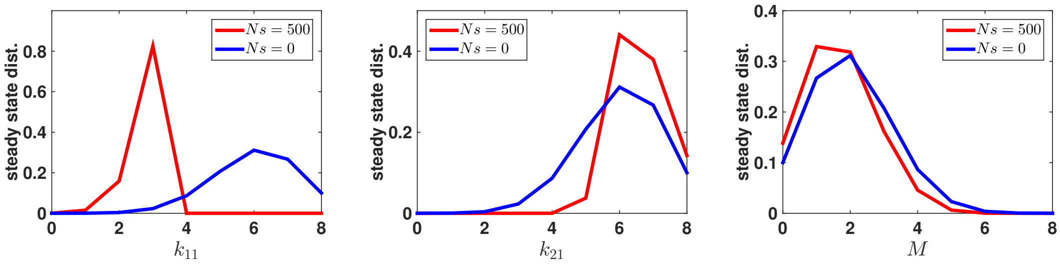

(a) (b) (c)

Supplementary Figure 8: **The steady state distributions of match, M, between TF consensus sequences when there is selection on the TFs to diverge is very similar to the neutral distribution.** We present analytically calculated steady state distribution of k_{11} (a), k_{21} (b) and M (c) for $Ns = 0$ (no selection, blue) and $Ns = 500$ (strong selection, red). The neutral distributions are always the Bernoulli distributions which here are peaked at $k = 6$ and $M = 2$. Selection to diverge biases the distribution to have a lower match than expected under neutrality. The difference between neutrality and selection becomes obvious only when looking at the distributions of k, the mismatches between BSs and TFs. Under neutrality the probability for match is low and the distribution is peaked around high mismatch values. When there is selection on the TF to remain functional it must preserve a low mismatch with at least one of the genes. Parameters: $L = 8$, $\epsilon = 3$, $C_0 = 3.269 \times 10^5$.

Probabilities of major macroscopic outcomes - losing a TF and specializing

In the main text we illustrate only the most probable macrostate for each parameter combination. Other macrostates are still possible, albeit with lower probability. Here we illustrate the probability to obtain either 'One TF Lost' or 'Specialize Both' macrostate at each parameter combination, as described in Supplementary Note 7. As shown in Suppl. Fig. 9, the steady state probability of specialization $Q_{\text{SS}}(\texttt{Specialize Both})$ is high at large Ns and intermediate ρ, and it decreases as selection strength decreases or signal correlation increases. The probability of having $Q_{\text{SS}}(\texttt{One TF Lost})$ at steady state is significant only when selection is not too weak and signals are highly correlated. Although for these parameter values it is the dominant macrostate its probability is only ~ 0.5, such that other macrostates are not negligible. In contrast, for parameter values where 'Specialize Both' dominates its probability is close to 1.

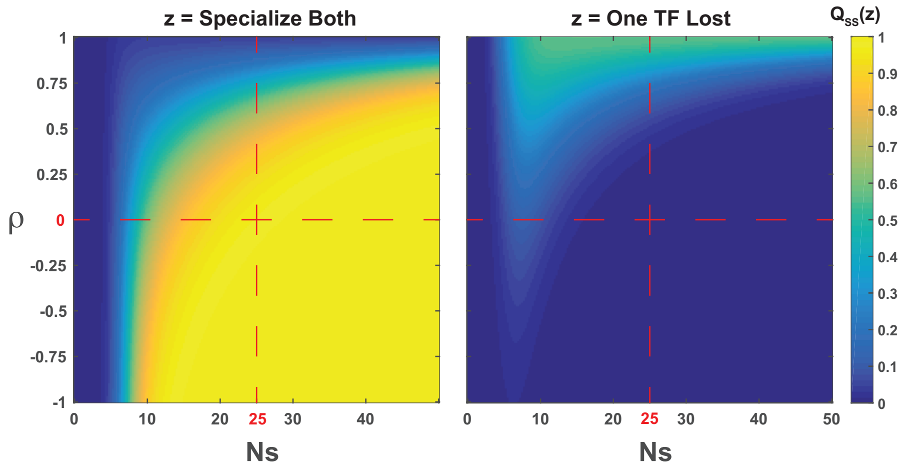

Supplementary Figure 9: **Steady state probabilities of 'Specialize Both' (left) and 'One TF Lost' (right) macrostates for different values of selection intensity Ns and correlation between the signals ρ.** The probability of either macrostate $Q_{\mathrm{SS}}(z)$ is illustrated using a color code (blue = 0, yellow = 1). Intersection of the red dashed lines denotes the baseline parameters values.

Asymmetric signal occurrence biases final outcomes

At the baseline parameters, we assume symmetry between the occurrences of the two signals, namely their frequencies $f_1 = f_2 = 0.5$, where $f_1 = \alpha_{10} + \alpha_{11}$ is the frequency of the first signal, and $f_2 = \alpha_{01} + \alpha_{11}$ is the frequency of the second. In the main text, in Fig. 3, we explored the role of signal frequency f_i, together with signal correlation ρ, while maintaining symmetry ($f_1 = f_2$). Here we explore the effect of asymmetry in signal occurrence ($f_1 \neq f_2$) on the final evolutionary outcomes and in particular on the probability to fully specialize. In Suppl. Fig. 10 we plot the most probable macrostate as a function of the signal frequencies f_1, f_2 for different values of selection intensities Ns when the signals are uncorrelated ($\rho = 0$); Suppl. Fig. 11 shows that at different ρ, f_1 and f_2 are constrained but the qualitative features of the plots are retained. When both signals are rare, $f_1, f_2 \ll 1$, `No Regulation` macrostate dominates, as selection on both pathways is weak. When one of the signals is frequent while the other is rare, $f_1 \gg f_2$, only the frequently used pathway is maintained, and the dominant macrostate is `Partial`. Only when both signals are frequent and selection is not too weak, specialization occurs. Hence, a signal-gene pathway is maintained only if it is required often enough, and the threshold for this (boundary between `Partial` and `Specialize Both`) depends on selection strength Ns. As selection strength Ns increases, this threshold moves to lower f_1 and f_2. As the frequencies of both signals increase, the dominant macrostate `Specialize Both` is replaced by `Specialize Binding`, where sensing one signal is a good proxy for the other signal as well, and later by `One TF Lost` when one TF is sufficient to transduce both signals.

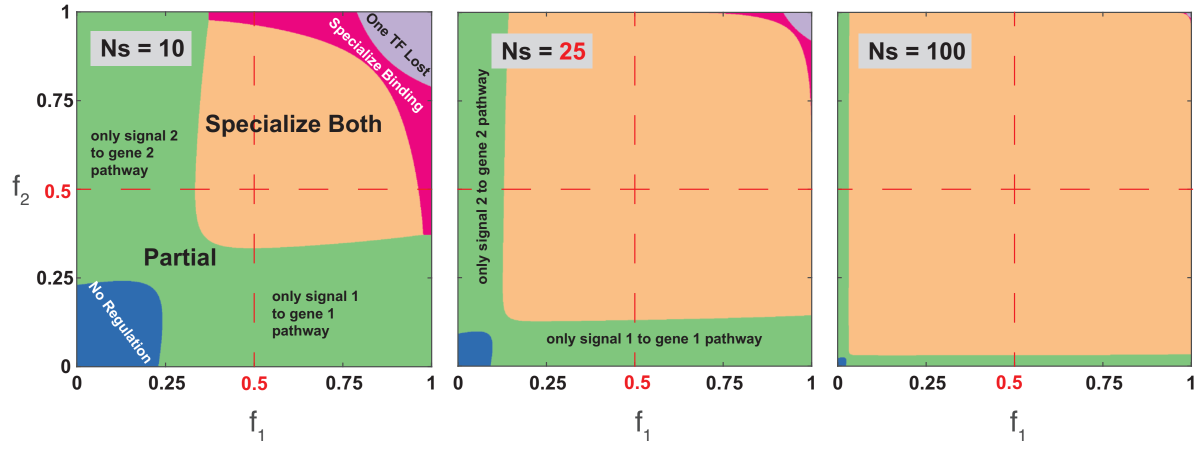

Supplementary Figure 10: **Under medium to strong selection, specialization occurs under a broad range of signal frequencies. Under weak selection specialization occurs only if signal frequencies are sufficiently high.** Phase plots of the most probable macrostate at steady state as a function of signal frequencies f_1 and f_2, at three different selection strengths $Ns = 10, 25, 100$. The intersection between the red dashed lines, $f_1 = f_2 = 0.5$, denotes the baseline parameters used anywhere else in this work.

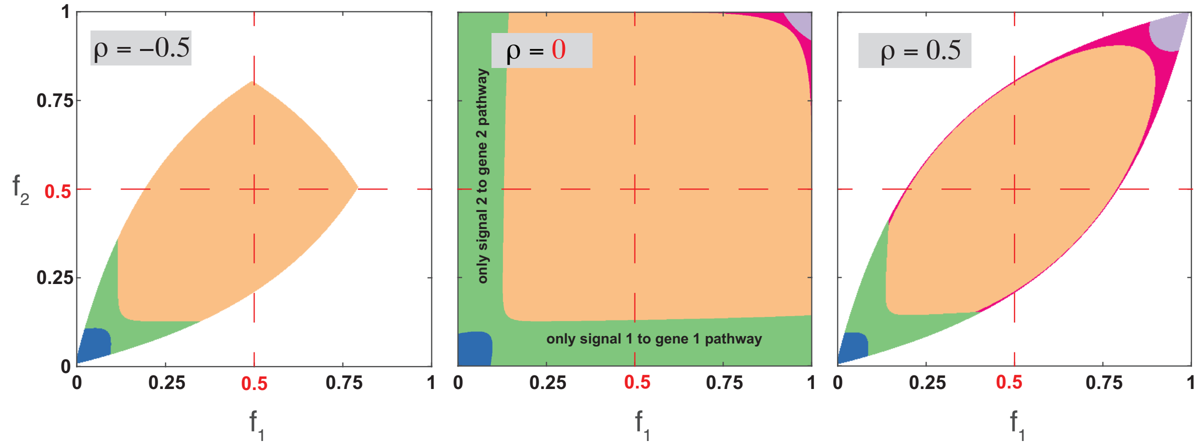

Supplementary Figure 11: **For different ρ, f_1 and f_2 are constrained, but the phase plots in the accessible region are similar.** Phase plots of the most probable macrostate at steady state (at $Ns =$ 25 and baseline parameters) as a function of signal frequencies f_1 and f_2, at three different intersignal correlation values, $\rho =$ -0.5, 0, 0.5. The white region of the plots denotes forbidden areas; ρ, f_1 and f_2 constrain each other and hence, not all (f_1, f_2) pairs are possible (see Eq. (S3)).

TFs as repressors

In the main text and in all the model variants in the SI, we assumed that TFs are activators. Here, we explore the case that TFs act as repressors. As described in Suppl. Fig. 12 the difference is in the mechanistic response of repressors to external signals compared to activators. In the absence of signal, repressor TFs are in their active state, where they can bind their binding sites and repress the corresponding genes. In the presence of signal, repressor TFs become inactive, avoid binding to their binding sites and consequently do not repress the corresponding genes. We assume

throughout a weaker penalty $\beta_X < 1$ on unnecessarily activating a gene compared to the higher penalty $\beta = 1$ on avoiding activation of a gene when needed, for either TF type. There is however an entropic difference between binding states realized by few TF-BS combinations and unbinding states realized by many such combinations and hence they can occur even neutrally. Consequently, the unequal penalties have different selective implications on activators and repressors: For activators there is a strong selection to bind (activate the gene when needed) and weak selection to avoid binding (cross-interactions inadvertently activating an unneeded gene). For repressors it is the opposite: strong selection to avoid binding (cross-interactions inadvertently repressing a gene that is needed), but weak selection to bind (repress when the gene is not needed). Due to the entropic difference, binding avoidance can occur even in the absence of selection, but for binding to occur significant selection is needed. This amounts to effective rescaling of selection pressures in the repressor case (with respect to the activator case). This result is demonstrated in Suppl. Fig. 13, where we plot the dominant macrostate at steady state as a function of Ns and ρ at baseline parameters (with $\beta_X = 0.5$) when the TFs act as repressors. Notice that this is mostly similar to Fig. 3 of the main text, where TFs act as activators, except for the rescaling of the x-axis (selection pressure). Hence transitions between macrostates: `No Regulation` to `Partial` to `Specialize Both` occur at larger Ns values compared to Fig. 3. In the special case that all penalties are equal $\beta_X = 1$, the activator and repressor cases provide the same results (right side of Suppl. Fig. 13).

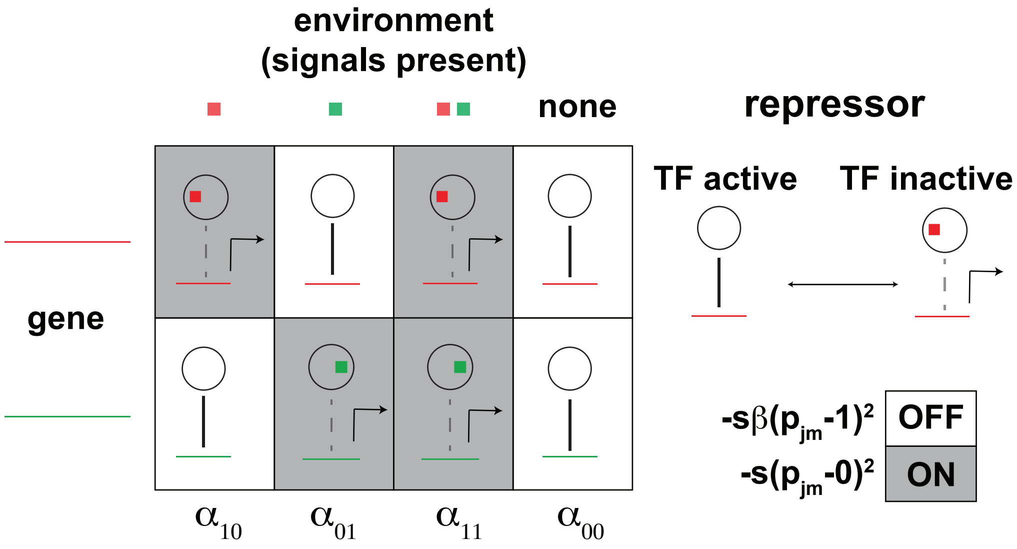

Supplementary Figure 12: **Optimal expression patterns and fitness contributions in different environments with repressor TFs.** When TFs act as repressors, the regulatory situation differs from the activator case (compare to Suppl. Fig. 1). With repressors, the genes are assumed to be active by default (grey background), unless repressed by a TF (white background). In contrast to the activator case, repressor TFs are active in the absence of a signal, consequently binding to their binding sites and repressing their target genes. In the presence of a signal, the TFs become inactive, and do not repress the genes (right scheme). In the table we list the optimal expression patterns of the two genes for all four signal combinations, and the repressor role in each case. In the bottom right we list the fitness penalties for both failure to activate and unnecessary gene activation, where $\beta \leq 1$.

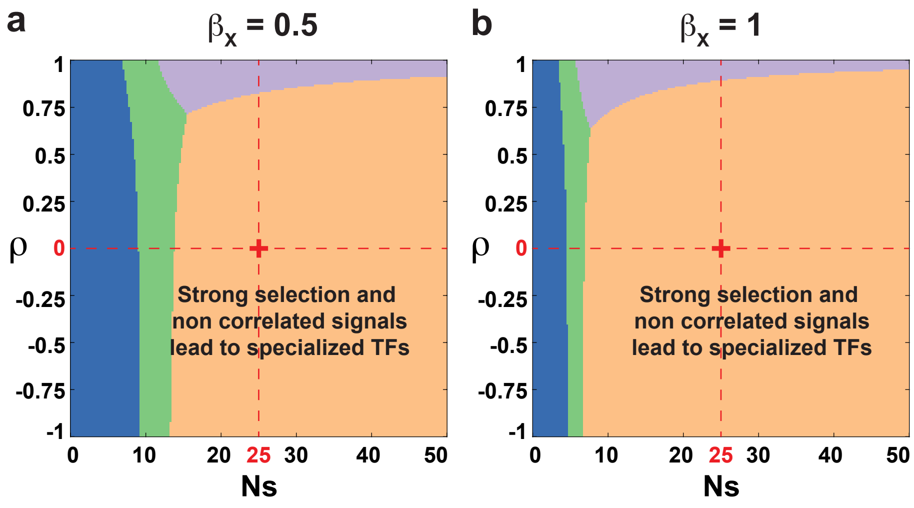

Supplementary Figure 13: **Dominant macrostate plots vs Ns and ρ when TFs act as repressors** (a) For $\beta_X = 0.5$ we find rescaling of the x-axis (selection intensity) with respect to the activator case. (b) For equal penalties on all deviations from the desired expression pattern $\beta_X = 1$, the evolutionary outcomes for repressors are equivalent to those obtained with activators (with $\beta_X = 1$ as well).

In Suppl. Fig. 14, we explore the role of signal frequency, f_i, on the dominant macrostate in the case of TFs acting as repressors. Note that this is a reflection, on the f_i axis, of the plot in the activators case (Fig. 3 of main text). At low signal frequencies, $f_i \approx 0$, the genes are required to be OFF together most of the time, and hence, one repressor TF can regulate both genes by always binding to their binding sites. This results in a dominance of the `One TF Lost` state. At high signal frequencies, $f_i \approx 1$, both genes are required to be ON together most of the time, and hence, repressor-BS binding occurs very rarely, thereby experiencing negligible selection pressure to maintain repressor-BS binding. Hence, the dominant state is that of `No Regulation`, where the repressor TFs do not bind to their binding sites, and hence, the genes are always ON.

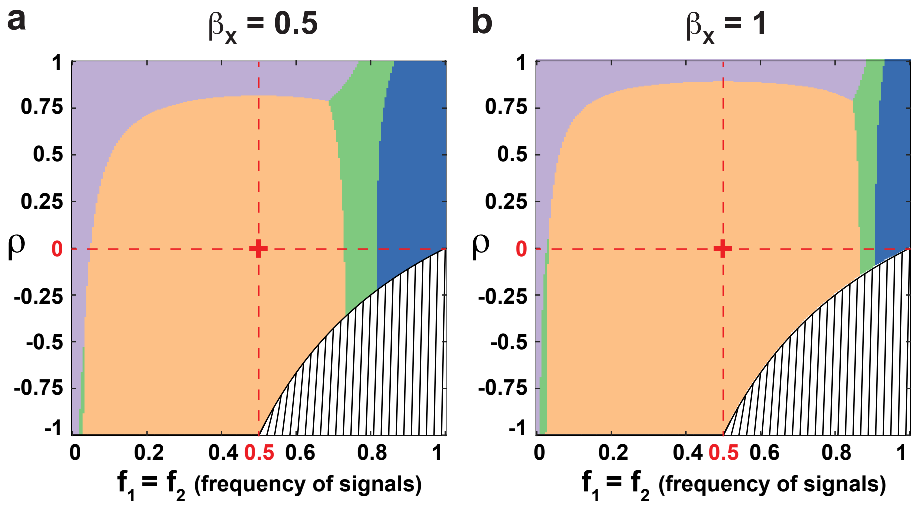

Supplementary Figure 14: **Dominant macrostate plots vs ρ and $f_1 = f_2$ when TFs act as repressors for (a) $\beta_X = 0.5$ and (b) $\beta_X = 1$.**

In Suppl. Fig. 15, we explore the effect of asymmetry in signal occurrence ($f_1 \neq f_2$) on the final evolutionary outcomes and in particular on the probability to fully specialize. We plot the most probable macrostate as a function of the signal frequencies f_1, f_2 for different values of selection intensities Ns when the signals are uncorrelated ($\rho = 0$); Suppl. Fig. 16 shows that the qualitative features of the plots are retained at different ρ values (not all f_1 and f_2 are possible, though). The principal difference from the activators case is that specialization now occurs at lower signal frequencies, with the `One TF Lost` state dominating at very low f_i, and `No Regulation` state domination at very high f_i.

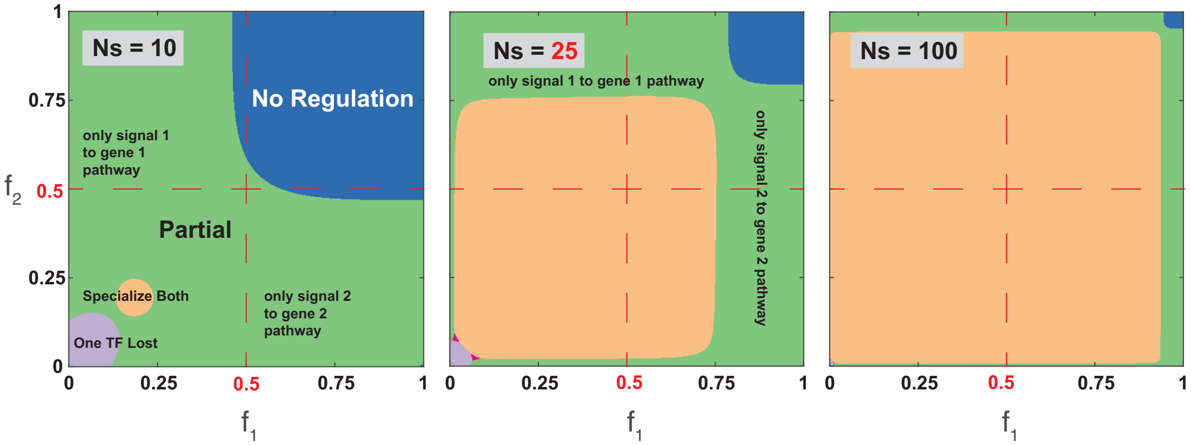

Supplementary Figure 15: **Under medium to strong selection, specialization occurs under a broad range of signal frequencies. For repressor TFs under weak selection, specialization occurs only if signal frequencies are low.** Plots show the most probable macrostate at steady state (for $\rho = 0$) as a function of signal frequencies f_1 and f_2, at three different selection strengths $Ns = 10, 25, 100$ when TFs act as repressors. The intersection between the red dashed lines, $f_1 = f_2 = 0.5$, denotes the baseline parameters used anywhere else in this work.

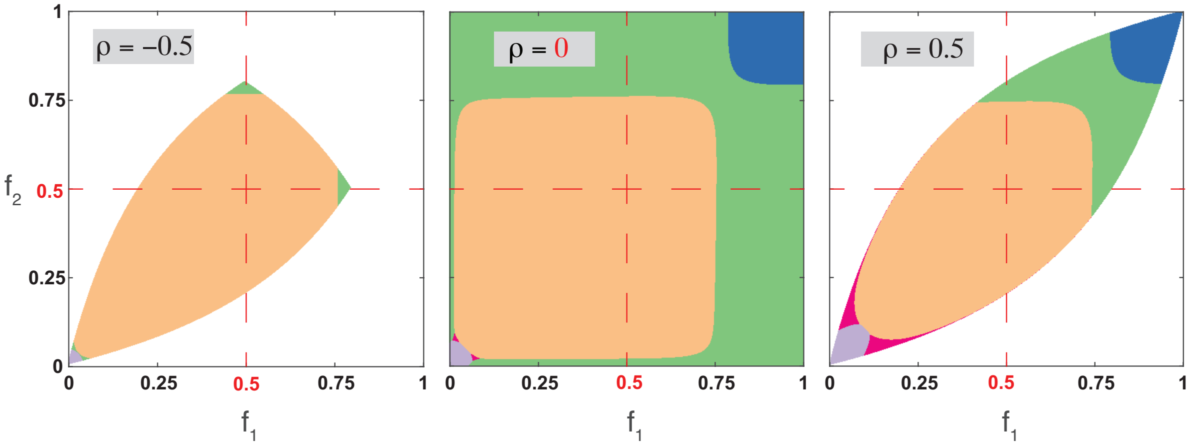

Supplementary Figure 16: **Most probable macrostate at steady state only weakly depends on the signal correlation ρ.** Plots show the most probable macrostate at steady state (at $Ns = 25$ and baseline parameters) as a function of signal frequencies f_1 and f_2, at three different signal correlation values, ρ = -0.5, 0, 0.5. The white region of the plots represents impossible parameter combinations; ρ, f_1 and f_2 constrain each other and hence, not all (f_1, f_2) pairs are possible for different ρ (see Eq. (S22)).

Supplementary Note 3 Role of β_X, the relative fitness penalty on crosstalk interactions

Transcription factors often bind weak secondary binding sites besides their primary target(s). This can lead to spurious activity of genes called crosstalk, i.e., deleterious activation of genes that does not happen via their primary regulatory pathway. For example, in our model a gene can be activated even if the signal to which it should respond is absent only because of (weak) binding of a transcription factor responding to another signal to its binding site. Previously, we studied the effect of crosstalk interference on gene regulation, and showed how it can place global constraints on the gene regulatory system [11]. Here, we explore the potential role of such crosstalk interactions in shaping the evolutionary trajectories of TF specialization.

The fitness of each reduced-genotype $x \in \mathcal{G}$ depends on the difference between the actual expression pattern the genotype generates and the ideal expression pattern as defined in Eq. (S2).

$$F(x) = -s \sum_j \sum_m \alpha_m \beta_{jm} (p_{jm} - p^*_{jm})^2. \tag{S11}$$

Here, β_{jm} weigh the penalties on different deviations from the desired expression level p^*_{jm}. In a certain environment m some genes should be active, $p^*_{jm} = 1$, while others should remain inactive, $p^*_{jm} = 0$. In our model, we allow for different penalties in either case. We penalize deviations from desired activity $p^*_{jm} = 1$ by setting $\beta_{jm} = 1$. We consider deviations from desired inactivity $p^*_{jm} = 0$ as less crucial and penalize them to a lesser extent $\beta_{jm} = \beta_X$, $\beta_X \in [0, 1]$. At the two extremes, if $\beta_X = 0$, no penalty on these crosstalk terms applies, while if $\beta_X = 1$, penalties on all deviations are equally important. In the main text, we used an intermediate value of $\beta_X = 0.5$. In this section we explore the role of β_X on the steady state distribution prior to and after TF duplication and on the evolutionary dynamics of specialization.

Steady state before duplication

A steady state distribution is attained before duplication, when only a single TF regulates all genes. In Suppl. Fig. 17 we illustrate the most probable macrostate prior to duplication for different values of cross-interaction penalties β_X. The macrostates possible before duplication are `Initial` (both genes regulated), `No Regulation` (none regulated) and some (but not all) variants of `Partial` - see Suppl. Fig. 17A for illustration. For $\beta_X \simeq 1$, the fitness penalty on mistakenly activating a gene is comparable to the fitness penalty on not fully inducing genes when needed, resulting in network configurations in which only one of the two genes is regulated (corresponding to `Partial` macrostate immediately after duplication for most $\rho < 0$). This is because, while configurations with only one gene regulated have one functional interaction and no crosstalk interactions, configurations with both genes regulated have two functional interactions and two crosstalk interactions. As β_X decreases, the selection against crosstalk interactions becomes weaker, resulting in configurations in which both genes are regulated (`Initial` macrostate immediately after duplication) even when $\rho < 0$.

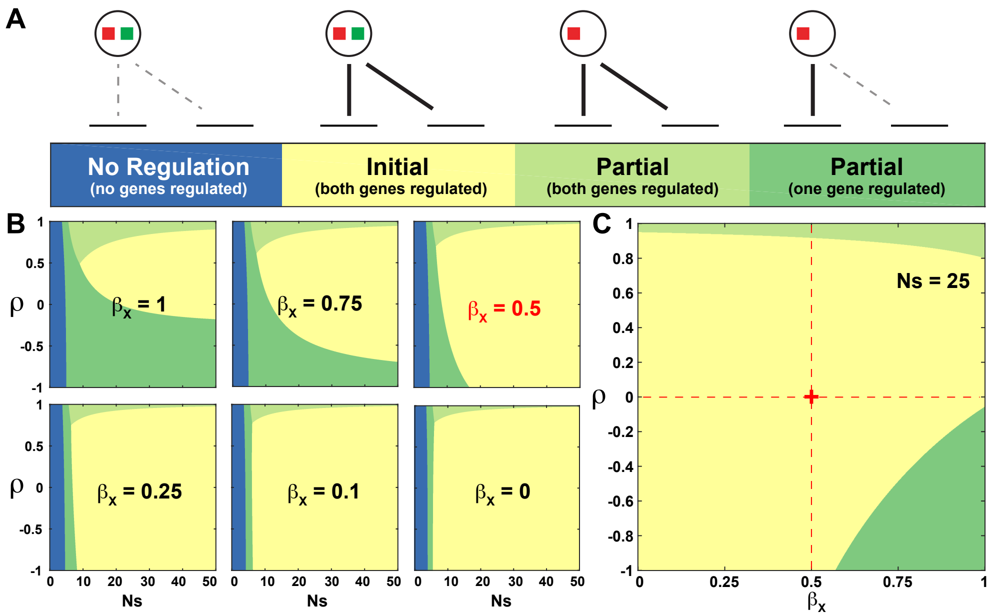

Supplementary Figure 17: **Dominant macrostate at steady state before duplication depends on β_X (crosstalk interaction penalty).** (A) Illustration of the different macrostates when only a single TF exists. Macrostates before duplication are defined in terms of the macrostate they would result in, if a duplication occurred on those genotypes. (B) Most probable macrostate at steady state before duplication, as a function of selection strength, Ns, and the correlation between the two external signals, ρ, for different values of β_X, the relative weight of fitness penalties corresponding to crosstalk interactions. (C) The most probable macrostate at steady state before duplication, as a function of β_X and ρ at $Ns = 25$.

Steady state after duplication

We proceed to observe the effect of varying β_X on the steady state after duplication, analogous to Fig. 3 of the main text where we assumed $\beta_X = 0.5$. In Suppl. Fig. 18, we show the phase plot of the most probable outcome of duplication at steady state for different values of β_X. The qualitative features of this phase plot are invariant to changes in β_X, as long as $\beta_X > 0$. For ρ not too close to 1, we obtain transitions from `No Regulation` to `Partial` and to `Specialize Both` as Ns increases. For large enough Ns, as ρ increases, there is a shift from `Specialize Both` to `One TF Lost`, via `Specialize Binding`, the width of which increases as β_X decreases. This is because there is reduced selection pressure on avoiding crosstalk interactions as β_X decreases. For small β_X, as ρ increases, it is sufficient that one of the TFs senses both signals while the TFs are still specialized in binding. As ρ increases even further, it is sufficient to have one TF mediating both pathways, marking the shift to the `One TF Lost` macrostate. These transitions occur very prominently for very small $\beta_X \approx 0$, where `One TF Lost` is the most probable outcome for all ρ values. Many models of duplication do not consider crosstalk interactions in their fitness function, and hence deal with the case of $\beta_X = 0$, making it important for comparison to our results.

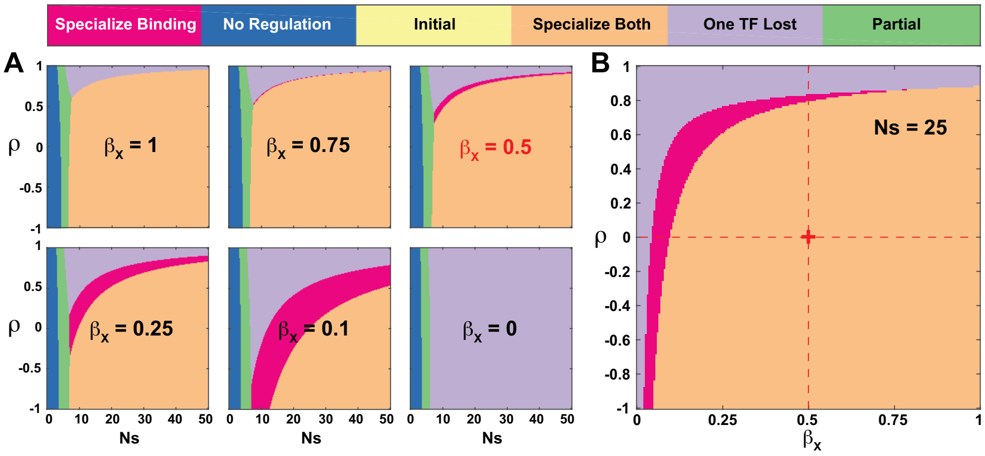

Supplementary Figure 18: **Dependence of steady state after duplication on β_X, the fitness penalty on cross-interactions.** (A) The most probable macrostate at steady state after duplication, as a function of selection strength, Ns, and the correlation between the two external signals, ρ, is plotted for six different values of β_X. (B) The most probable macrostate at steady state after duplication, as a function of β_X and ρ at $Ns = 25$. An increase in β_X has a a similar effect to an increase in selection intensity on all interactions by varying Ns.

Evolutionary dynamics

To understand how β_X affects the evolutionary dynamics of specialization, we first obtained the dynamics of the most probable macrostate as a function of ρ and β_X for fixed selection intensity $Ns = 25$ (baseline parameters). In Suppl. Fig. 19, we plot a few snapshots of the phase diagram of the most probable macrostate at different time-points after duplication, starting from $t = 0$ (immediately after duplication), to $t = \infty$ (steady state after duplication). Specialization is faster for smaller ρ because the fitness benefit of eliminating crosstalk interactions is larger. Likewise, specialization is faster for larger β_X as the selection strength against crosstalk interactions is higher. A huge region of the (β_X, ρ) plane corresponding to small β_X or large ρ, most of which starts at `Initial` and specializes via the slow pathway of `One TF Lost`.

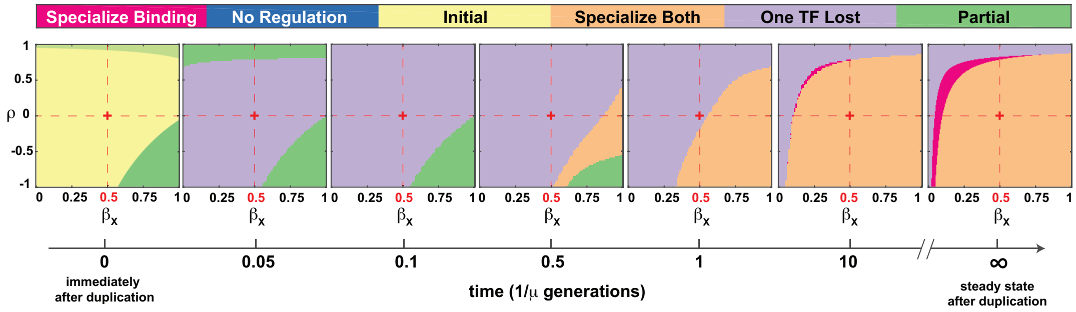

Supplementary Figure 19: **Snapshots of the most probable macrostate at different time-points post-duplication.** The most probable macrostate as a function of signal correlation, ρ, and β_X, the relative weight of fitness penalties corresponding to crosstalk errors, for $Ns = 25$. The left-most phase plot corresponds to the time-point immediately after duplication, and the right-most phase plot corresponds to the steady state after duplication. For other parameters, the baseline values have been used. $\beta_X = 1$ corresponds to equal-magnitude selection strengths on functional as well as crosstalk interactions; $\beta_X = 0$ corresponds to no selection against crosstalk interactions. In the main text, we choose $\beta_X = 0.5$ as the baseline parameter value.

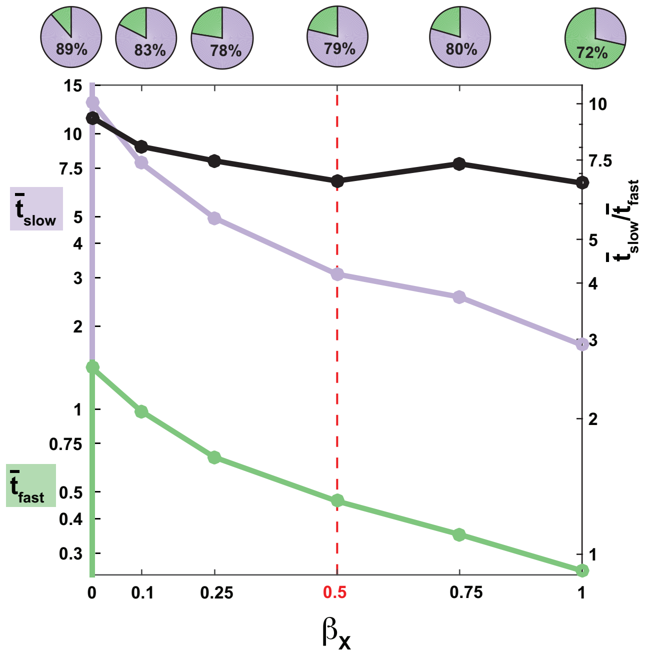

Supplementary Figure 20: **How do the slow and fast pathways to specialization depend on β_X?** For large β_X, the time to specialization shortens for all pathways and the fraction of trajectories to specialization taken via fast pathways (through `Partial` macrostate) increases. Pie charts illustrate the fraction of slow (lavender) and fast (green) trajectories for different values of β_X. The black line (right y-axis) shows the ratio between average specialization times, which does not significantly change with β_X. For other parameters, the baseline values were used. $\beta_X = 1$ corresponds to equal-magnitude selection strengths on functional as well as crosstalk interactions; $\beta_X = 0$ corresponds to no selection against crosstalk interactions. In the main text, we choose $\beta_X = 0.5$ as the baseline parameter value.

Next we sought to understand which pathways are taken towards specialization for different β_X by running many repeats of simulations at each β_X. For each β_X, we found the most probable

genotype at steady state before duplication and ran many repeats of the simulation starting from that genotype. In Suppl. Fig. 20, we explore the dependence on β_X of fraction of the two pathways to specialization (slow via `One TF Lost` and fast via `Partial`), and also the corresponding times to specialization. First of all, specialization becomes quicker as β_X increases from 0 to 1. This is because stronger selection against the crosstalk interactions eliminates them faster. Secondly, the relative speed of the fast pathway (compared to the slow pathway) depends only very weakly on β_X. Thirdly, about 80% of trajectories follow the slow pathway, and this depends only very weakly on β_X, till $\beta_X = 0.75$. In contrast, for $\beta_X = 1$, the fast pathways via `Partial` become predominant. This occurs because the steady state before duplication (which acts as the initial condition for the trajectories) flips from `Initial` to `Partial`.

Supplementary Note 4 Evolutionary dynamics

Evolutionary trajectories between the post-duplication unspecialized configuration ('Initial') to full specialization ('Specialize Both' macrostate) are multi-step processes that require several mutations and transiently pass through various macrostates. Here we describe the various trajectories for this functional transition.

Evolutionary pathways - first model variant

In Suppl. Fig. 21 we detail the different pathways to specialization. The pathways proceeding via `One TF Lost` are slow compared to the pathways proceeding via `Partial` which are faster. The mutation initiating the process in all pathways is neutral and hence the ratio between r_S (signal sensing domain mutations rate) and r_{TF} (TF mutation rate) determines which pathway is more likely to occur - see Suppl. Fig. 22.

Along the slow `One TF Lost` pathway, typically, first a TF consensus sequence mutation occurs that weakens the binding of one TF to both binding sites. Once binding is lost, further mutations cause the TF consensus sequence to neutrally drift away. Meanwhile, the lost TF gains a sensing mutation such that it senses only one of the two signals. Next, a BS mutation in one of the binding sites flips its TF preference such that the system moves into `Specialize Binding` macrostate. This is a beneficial mutation as one of the signal-BS pathways becomes specific. This involves evolving a TF-BS link essentially from scratch; the lost TF consensus sequence is a random number of mismatches away from the binding site sequence, and the beneficial BS mutation can occur only when the TF consensus sequence, by chance, becomes close enough to the BS sequence. From `Specialize Binding`, another beneficial sensing mutation leads the system to full specialization (BS and signal).

There are multiple routes in the `Partial` pathway. In one of the routes, first a neutral TF consensus sequence mutation occurs such that the TF loses binding to only one of the two binding sites resulting in `Partial` macrostate. This is different from the first mutation in `One TF Lost` pathway where the TF loses binding to both binding sites. From here, a sensing domain mutation specializes one of the signal-BS pathways, making this mutation beneficial. Further, a neutral BS mutation brings the system to `Specialize Binding`, from where a beneficial sensing domain mutation leads the system to specialization.

In the second and third routes via the `Partial` macrostate, first a neutral sensing domain mutation occurs. Next, either a beneficial TF consensus sequence mutation can bring the system onto

the previous route or if the sensing domain mutation rate is high, another neutral sensing domain might occur first. From here, a beneficial TF consensus sequence mutation and a beneficial BS mutation again lead to full specialization.

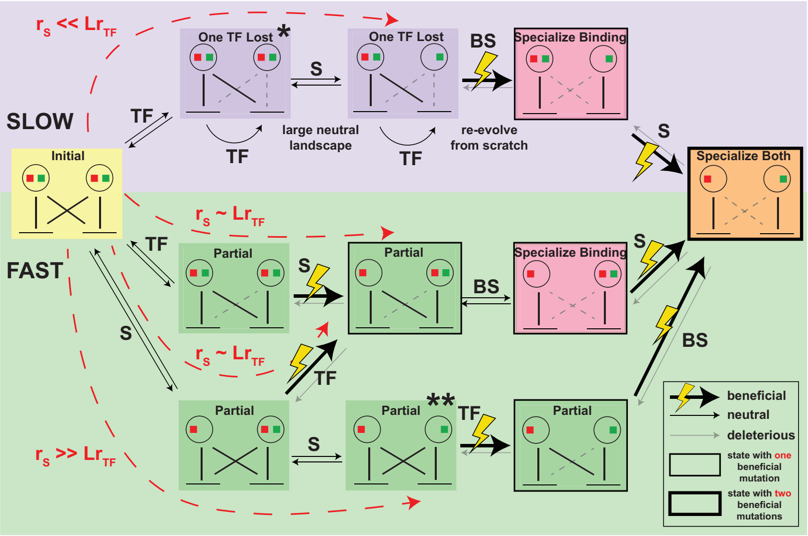

Supplementary Figure 21: **Pathways to specialization differ in the order and nature of mutations.** Here we detail the various mutations occurring along the different pathways to specialization. For each mutation, we show the type of mutation (in text on the arrows): TF consensus sequence mutation (TF), binding site sequence mutation (BS), TF signal sensing domain mutation (S) and whether it is beneficial, (nearly) neutral or deleterious (style of the arrows). We also illustrate the macrostates along each pathway using the same color code in the background as in the main text. The number of beneficial mutations in each macrostate relative to the `Initial` macrostate is depicted by box style (see legend). Text in red indicates the conditions on mutation rates that favor the different pathways. Note that from the `One TF Lost` state marked with a star, the "lost" TF can actually take up new functions (by sensing and binding to signals and binding sites other than those considered in our model), leading to "neo-functionalization". Also, the `Partial` state marked with two stars acts as the initial condition in the alternative model variant, with the TFs already specialize in signal sensing immediately post-duplication.

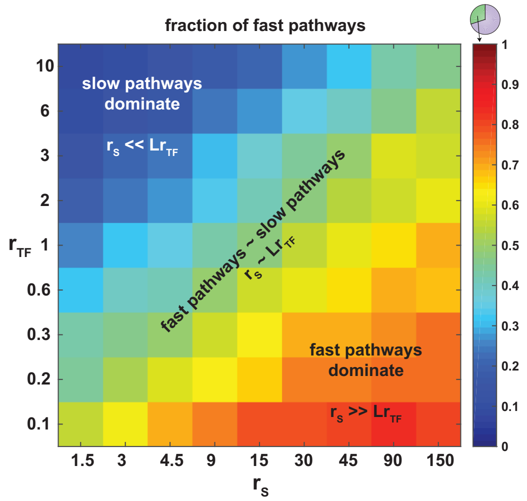

Supplementary Figure 22: **The ratio between r_S and r_{TF} determines the dominant pathway.** We plot the fraction of fast `Partial` pathways as a function of r_S (signal sensing domain mutation rate) and r_{TF} (TF mutation rate). Other parameters remain at their baseline values (see Supplementary Note 1). Color code denotes the fraction of fast pathways (specialization is reached via 'Partial' intermediate state).

Evolutionary pathways - second model variant

The second model variant (see Supplementary Note 1) assumes that immediately post-duplication, TFs are expressed at different times (or are already specialized with respect to their signal sensitivity), and that this is fixed for the rest of the evolutionary time. This `Partial` macrostate is marked by two stars in the pathway schematic Suppl. Fig. 21. In this setting, selection to specialize starts with a phase of fast diversification where each pair of TF-BS mutates (in orchestrated manner) to diverge from the other. The fitness benefit in diversification is large at the beginning when the TFs are identical, but diminishes the more distinct they become. This is illustrated in Suppl. Fig. 21 by the two TF and BS beneficial mutations that lead to specialization. After specialization, further TF diversification proceeds as a nearly neutral process, and hence occurs more slowly. These two phases, the fast adaptive one followed by the slow nearly-neutral one, are illustrated in Suppl. Fig. 23.

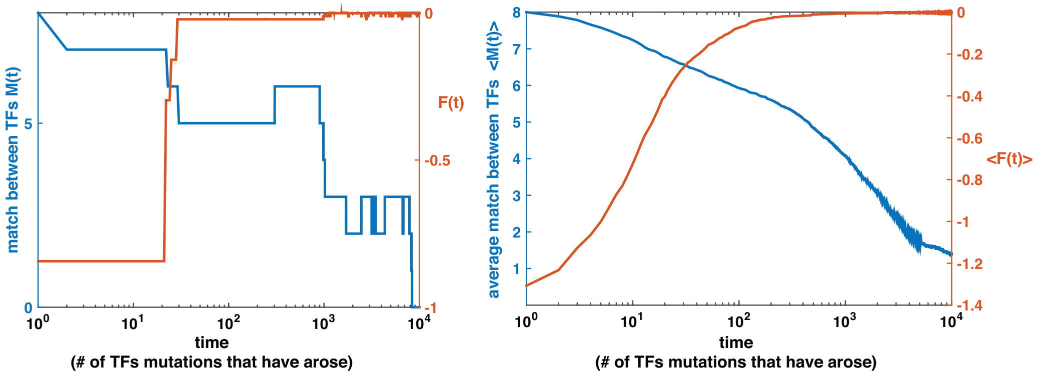

(a) (b)

Supplementary Figure 23: **In the second model variant (TFs specialized in the signal sensitivities or expression times immediately post-duplication) a significant proportion of evolutionary time is spent in neutral evolution phase.** Selection only works in the beginning of the evolutionary trajectory to exert diversification, but a significant part of TF diversification occurs almost neutrally with only a modest fitness benefit involved. We illustrate dynamical trajectories of the match between TFs, M, and the fitness, F, obtained in stochastic simulations. (a) shows a single trajectory and (b) shows an average over 400 independent repeats of the simulation. Each time unit is a simulation iteration in which a mutation in one of TFs occurs, but does not necessarily fix (see Supplementary Note 7).

Time to specialization

In Suppl. Fig. 24, we plot the average time to specialization via slow and fast pathways for various values of L, r_{TF} and r_S. The ratios of these times are plotted in Fig. 4 of the main text. Increasing either mutation rate by changing r_{TF} or r_S speeds up specialization via both pathways because mutations occur faster. Increasing L slows down the slow `One TF Lost` pathway because of an increase in size of the neutral landscape; strikingly, increasing L does not lengthen the fast pathway through `Partial` states.

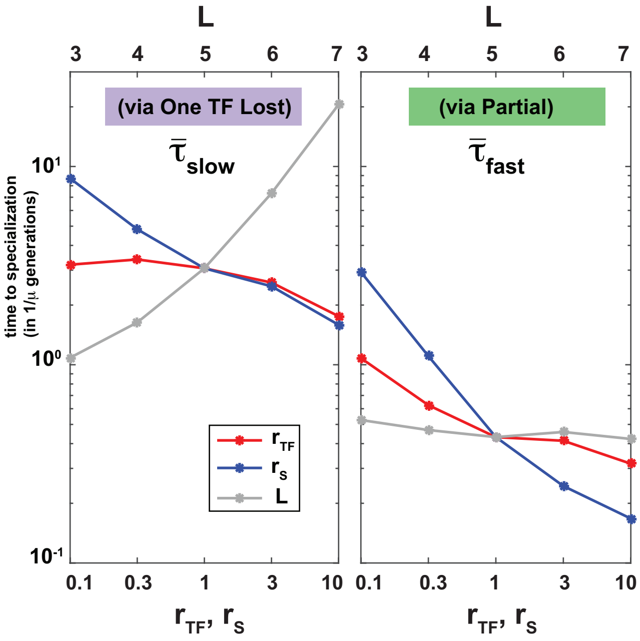

Supplementary Figure 24: **Time to specialization via different pathways for different parameters.** We plot the mean times to specialization, $\bar{\tau}_{slow}$ and $\bar{\tau}_{fast}$, via the slow (left panel) and the fast (right panel) pathways, while varying L (grey curve, top axis), r_{TF} (red, bottom axis) and r_S (blue, bottom axis) separately. Other parameters remain at their baseline values. We find opposite dependence of the time to specialize on the binding site length L in the distinct pathways. While for pathways going via 'One TF Lost' (left panel) time increases with L due to increase in the sequence space, it mildly decreases with L for pathways going via 'Partial'. For all pathways time decreases if mutation rates increase.

Supplementary Note 5 Multiple genes regulated by each TF post-duplication

Steady state after duplication

Transcription factors often regulate multiple downstream genes, rather than one gene post-duplication, as we considered so far. Here we generalize our analysis to account for a general number of genes, n_G. The steady state distribution in the general case is

$$P(M, \{k_{ij}\}, \{\sigma_i\}) = P_0(M, \{k_{ij}\})P_0(\{\sigma_i\})\exp(2NF), \tag{S12}$$

where P_0 is the neutral distribution and F is the fitness of the reduced-genotype. First, we need to account for the neutral distribution P_0 (entropic factor). This is straightforward, because for given TF consensus sequences, the probability that a particular binding site j has mismatch values (k_{1j}, k_{2j}) is independent of the state of other binding sites. Thus, we can simply factor out the probabilities for different genes:

$$P_0(M, \{k_{ij}\}, \{\sigma_i\}) = P_0(\{\sigma_i\})P_0(M)\prod_j P_0(k_{1j}, k_{2j}|M), \tag{S13}$$

where j enumerates the genes.

Second, we need to take care of the adaptive (energy) factor $\exp(2NF)$ in the general case. Because $F = \sum_j F_j$ is linear in terms of contributions F_j from each gene j, $\exp(2NF)$ factorizes into $\prod_j \exp(2NF_j)$. Hence, we have

$$P(M, \{k_{ij}\}, \{\sigma_i\}) = P_0(M) P_0(\{\sigma_i\}) \prod_j P_0(k_{1j}, k_{2j}|M) \exp(2NF_j). \tag{S14}$$

Now, for $\langle M \rangle$, we have,

$$\begin{aligned} \langle M \rangle &= \sum_{\{k_{ij}\}, M, \{\sigma_i\}} M P(M, \{k_{ij}\}, \{\sigma_i\}) \\ &= \sum_{\{\sigma_i\}} P_0(\{\sigma_i\}) \sum_M M P_0(M) \prod_j \sum_{k_{1j}, k_{2j}} P_0(k_{1j}, k_{2j}|M) \exp(2NF_j) \\ &= \sum_{\{\sigma_i\}} P_0(\{\sigma_i\}) \sum_M M P_0(M) \prod_j \langle \exp(2NF_j) \rangle_{P_0(\{k_{ij}\}|M)}. \end{aligned} \tag{S15}$$

$\langle \exp(2NF_j) \rangle_{P_0(\{k_{ij}\}|M)}$ can be calculated for each gene j separately. We consider n_G downstream genes split into two sets of size a and b ($n_G = a + b$), such that a genes should respond to the first signal and b genes respond to the second signal. We write this as $a + b$ schematically in the figures. For the main model, we had $a = b = 1$.

We find that the steady state distribution of M, the match between the two transcription factors, is independent of the number of downstream genes - see Suppl. Fig. 25.

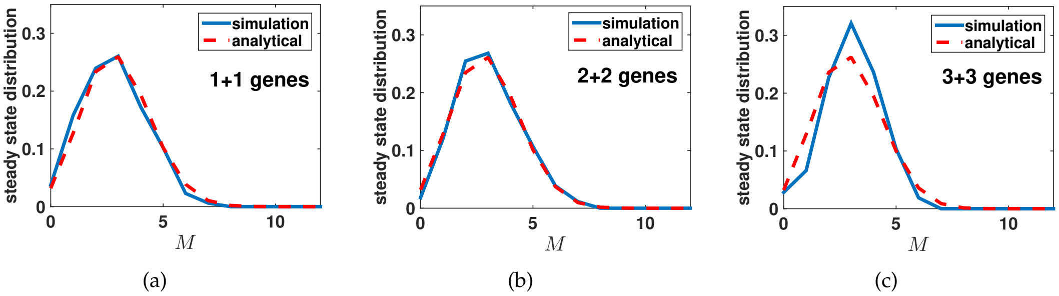

Supplementary Figure 25: The steady state distribution of M, the match between TF consensus sequences, is independent of the number of downstream genes regulated by these TFs. We present the analytically calculated steady state distribution and stochastic simulation results for $a+b$ =1+1, 2+2 and 3+3 downstream genes. Simulation steady state is the distribution obtained after 50,000 generations (1+1, 2+2 genes) or 150,000 generations (3+3 genes). Parameters: $L = 12$, $Ns = 500$, $\epsilon = 3$, $C_0 = 3.269 \times 10^5$, $r_{TF} = 0.02$ (TF mutation rate is 50 times lower than the BS mutation rate).

Evolutionary dynamics

Frustration of fitness landscape

Each TF needs to simultaneously regulate a subset of the genes while avoiding regulation of the remaining ones. This increasing number of constraints, relative to the $n_G = 2$ case, incurs a diminishing number of feasible evolutionary trajectories. The fitness change due to a TF consensus sequence mutation is assessed according to its effect on the binding affinities of this TF with all existing genes. Hence, for each TF, as n_G increases, the number of constraints also increases. This

limits the number of possible substitutions a TF can access via fewer beneficial and neutral mutations. In contrast, for each binding site, the number of constraints does not change because it is only constrained by the two TFs and not by other binding sites. To demonstrate how extra constraints arising for $n_G > 2$ genes affect evolutionary trajectories, we classified in Suppl. Fig. 26 the effects of all TF mutations on fitness for various numbers of downstream genes $a + b$.

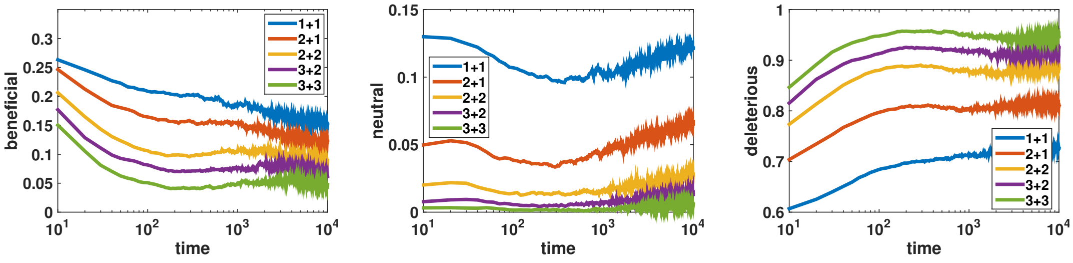

(a) (b) (c)

Supplementary Figure 26: **The fitness landscape becomes more frustrated when $n_G > 2$ (i.e., when each TF post-duplication regulates more than 1 gene).** At every time point in the stochastic simulation we analyze all possible TF consensus sequence mutations and classify them according to their effect on fitness as beneficial (a) neutral (b) or deleterious (c). With increasing number of downstream genes, $n_G = a + b$, regulated by each TF (different curve colors, see legend), the fractions of beneficial and neutral mutations decrease and the fraction of deleterious mutations increases. This is because TFs become more constrained as n_G increases, resulting in fewer potential mutations that are beneficial or neutral.

With increasing numbers of downstream genes, evolutionary trajectories are more often stuck in local fitness peaks. We demonstrate this effect in Suppl. Fig. 27, where we classified at each time point in the simulation all possible TF mutations, and determined that a particular point is a fitness peak if all possible TF mutations from that point are strictly deleterious. Evolution can still continue thanks to the binding sites mutations which are much less constrained.

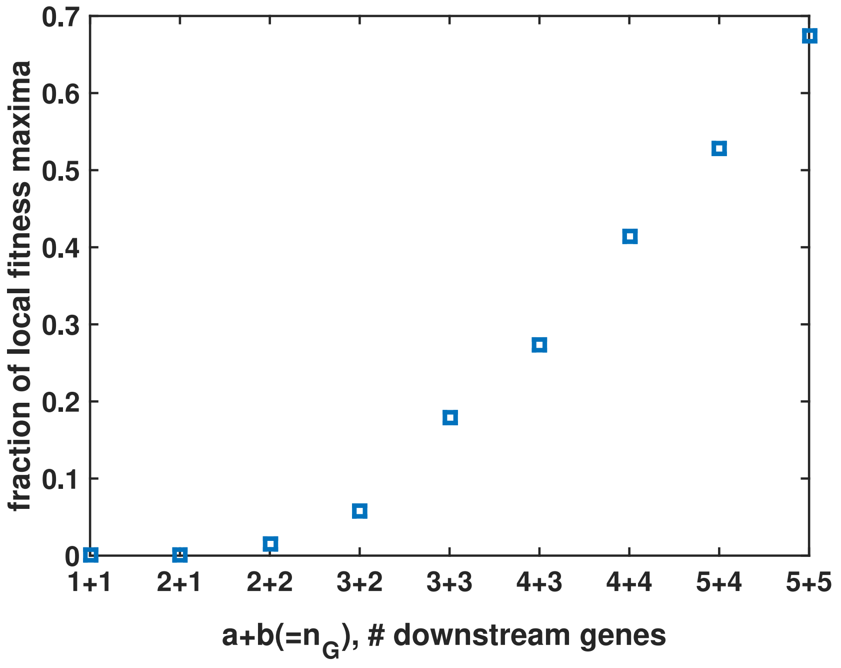

Supplementary Figure 27: **The adaptive landscape of TFs becomes more rugged the more genes they regulate.** We classify all possible TF mutations according to their fitness effect as beneficial, deleterious or neutral. If at a certain time point all mutations of both TF are strictly deleterious, this indicates a local fitness peak. A way out of such a peak, if there is one, proceeds by means of BS mutation(s), following which the TF can evolve further. The figure illustrates simulation-based statistics of the fraction of time points in which such fitness peaks are encountered for different n_G, split (un)equally, $n_G = a + b$, between the TFs (indicated on x-axis). Clearly, the more genes a TF needs to regulate, the more constrained it is, and the fewer are the trajectories it can take. The fraction of local fitness maxima depicted in the plot were obtained by sampling the fitness landscape along typical evolutionary trajectories, and hence does not reflect the entire fitness landscape. Each point is an average over 160,000 points (400 independent simulation repeats, 4000 time points sampled at a uniform interval between t=6000-10,000 when the dynamics is already nearly neutral (see Supplementary Note 7 for details). Parameters: $L = 8$, $Ns = 100$, $C_0 = 3.269 \times 10^5$, $\epsilon = 3$, $\beta_X = 1$.

Evolutionary pathways

The pathways to specialization in the case of multiple regulated genes are more complex than those described in Supplementary Note 4 for $n_G = 2$. The primary difference is that for $n_G > 2$ some pathways involve fitness valley crossings, where there is a chance of being stuck on local fitness peaks/plateaus. Hence, these paths take longer times to specialize. The following are the main pathways that are depicted in Suppl. Fig. 28. The first proceeds via `One TF Lost` macrostate while the other pathways proceed only via `Partial` configurations.

1. The first pathway involves the `One TF Lost` macrostate, where as before one TF does not bind to any binding site. Evolving a TF-BS link to this TF entails a random walk on a neutral landscape and essentially involves regulatory evolution from scratch. After gaining a TF-BS link from a BS mutation, the system ends up on a local fitness plateau (marked with a red box in Suppl. Fig. 28) in the `Partial` state. This is because the "lost" TF (second TF in the figure) has considerably diverged from the first TF yet has specialized only for some, but not all, of

the genes associated with the green signal, but not for all of them. All of the TFs and BSs are constrained to maintain match beyond some minimal level.

Hence specialization can only occur if one of the strong TF-BS links weakens. Such weakening decreases the fitness, and hence incurs crossing a fitness valley. This pathway is consequently very slow.

2. The remaining pathways do not involve `One TF Lost` macrostate and go only via `Partial` macrostate. In the second pathway, first, a TF consensus sequence mutation and a signal sensing mutation (either can occur first) lead the system to a `Partial` state with some of the signal-BS pathways specialized. Then, an additional TF consensus sequence mutation pushes the TFs further apart. This, together with BS mutations, brings the system to the local fitness plateau (in the `Partial` macrostate) described in the previous pathway. This pathway is also slow, because of the fitness valley crossing described above.

3. In the third pathway also, first, a TF consensus sequence mutation and a signal sensing mutation (either can occur first) lead the system to a `Partial` state with some of the signal-BS pathways specialized. From here, no additional TF consensus sequence mutations occur that push the TFs away. Hence, there are paths for the BSs to realign their binding preferences (to the other TF) such that fitness is always maintained and does not involving crossing any fitness valleys. Hence, this pathway is fast.

4. In the fourth and the fifth pathways, the first two mutations are signal sensing mutations that specialize the TFs' signal sensing domains. From here, a TF mutation and subsequent BS mutations can specialize without going through fitness valleys. Hence, this is a fast pathway. For a given genotype (specifying the TF and BS sequences), this fourth pathway is either possible or not. If it is not possible, then the only resort is the fifth pathway.

5. The fifth pathway comes into play when the fourth pathway is not possible. This happens when any TF mutation loses some signal-BS pathways, hence dropping the fitness considerably. The TFs cannot diverge at all, and this involves crossing a fitness valley. Hence, this is a slow pathway.

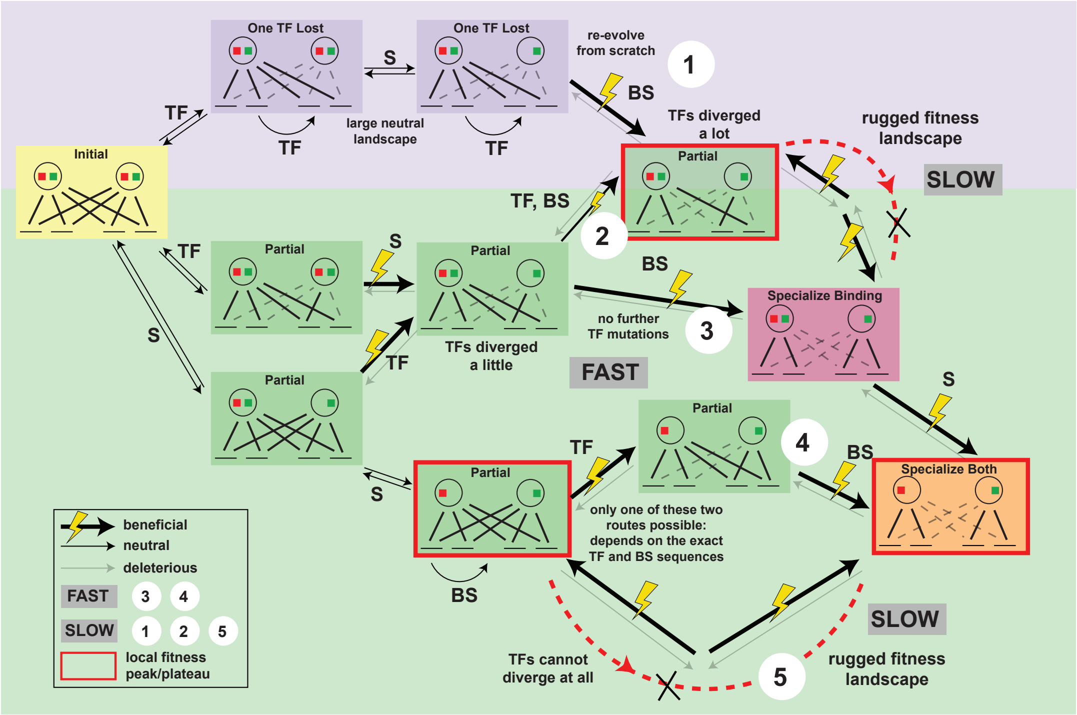

Supplementary Figure 28: **Different pathways to specialization vary in the order and nature of mutations, and might have to cross a rugged fitness landscape for $n_G > 2$.** Here we show in detail the various mutations that occur along the different pathways (marked with numbers inside white circles) to specialization. For each mutation, we show the type of mutation (text on the arrows): TF consensus sequence mutation (TF) or binding site sequence mutation (BS), TF signal sensing domain mutation (S) and whether it is beneficial or (nearly) neutral or deleterious (style of the arrows, see legend). We also depict the macrostates along each pathway graphically, and mark local fitness peaks/plateaus with red boxes. In red dotted curved lines, we denote parts of the pathways which involve a fitness valley and hence, are very difficult to cross. Routes not involving any fitness valleys (numbered 3 and 4) are fast, while those involving a fitness valley (numbered $1, 2$ and 5) are slow.

Time to specialization

By running simulations, we calculate the time to specialization for different values of $n_G > 2$ (total number of downstream genes) via the different pathways described in the previous section. Specifically, we calculate the time to specialization, τ_1, via the `One TF Lost` pathway (pathway 1), τ_{3+4}, via the fast `Partial` pathways (pathways 3 and 4), and, τ_{2+5}, via the slow `Partial` pathways (pathways 2 and 5). We also calculate the fractions of these pathways. These are shown in Suppl. Fig. 29. The slow `Partial` pathway (numbered 2 and 5) is absent for $n_G = 2$. The fast `Partial` pathway (numbered 3 and 4) does not involve crossing any fitness valleys, and hence the time to specialization via this pathway decreases with increasing Ns for all n_G. The time to specialization via the slow `One TF Lost` pathway (numbered 1) decreases with increasing Ns for $n_G = 2$, and so does not involve crossing fitness valleys. For $n_G > 2$, the time to specialization via both the slow `One TF Lost` pathway and the slow `Partial` pathway increases as Ns increases. Both these pathways for $n_G > 2$ involve crossing fitness valleys. With increasing n_G, the fractions of the fast `Partial` pathway and slow `Partial` pathway increase at the expense of the slow `One TF Lost` pathway.

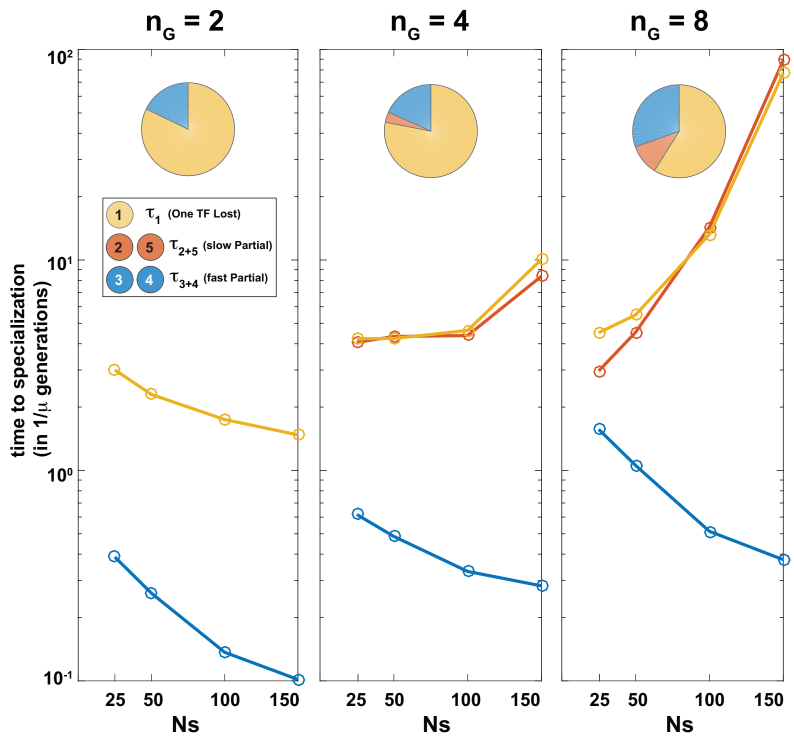

Supplementary Figure 29: **Times to specialization via different pathways for various numbers of downstream genes.** Shown are the times to specialization via different pathways as a function of Ns for different values of n_G. We plot the times for the slow `One TF Lost` pathway (numbered 1, yellow), the slow `Partial` pathway (numbered 2 and 5, red), and the fast `Partial` pathway (numbered 3 and 4, blue). Plotted as pie charts also are the fraction of various pathways for different n_G values as pie charts; these fractions depend only very weakly on Ns. In general, the higher the n_G, the larger the fraction of fast trajectories (3 and 4) and the longer the time needed to specialize. Pathways whose time lengths with Ns, which are the slow `Partial` pathway (red) and the `One TF Lost` pathway (yellow) for $n_G > 2$, involve crossing fitness barriers.

Supplementary Note 6 Promiscuity-promoting mutations

So far we considered the "mismatch-energy model" for TF-BS specificity, where each position in the TF and the binding site contributed equally to the total binding energy, depending on whether the position has a mismatch between the TF consensus sequence and the BS sequence. Let the TF consensus sequence be s^* and the binding site sequence be s, both of length L. In general, we have

$$E = \sum_i E_i \tag{S16}$$

where i runs over all the positions of the binding site. For each specific position i, the contribution is $E_i = 0$ if $s_i = s_i^*$ (match) and $E_i = \epsilon$ if $s_i \neq s_i^*$ (mismatch).

Experiments on TF-BS specificity, however, suggest that some TF (and binding site) positions dominate while others only have minor energetic contributions. In this section we study a simple generalization of the mismatch-energy model, where we allow for two levels of contribution: some positions are specific (favor a unique nucleotide) and have large energetic contribution while others are non-specific or promiscuous (all nucleotides are equally favorable) and have a smaller energetic contribution. For each specific position i, the contribution E_i is, as in the mismatch-energy model,

ϵ if there is mismatch between the TF consensus sequence and the BS sequence in that position, and 0 if there is a match. On the other hand, for each promiscuous position i, the contribution is $E_i = \epsilon_P$ (typically $0 \leq \epsilon_P \leq \epsilon$), independent of s_i. Hence, for a TF with $L_P < L$ promiscuous positions in total, and k mismatches in the remaining $L - L_P$ specific positions, the total binding energy would be $E = \epsilon_P L_P + k\epsilon$. The different possible energy levels for specific and promiscuous TFs are illustrated in Suppl. Fig. 30.

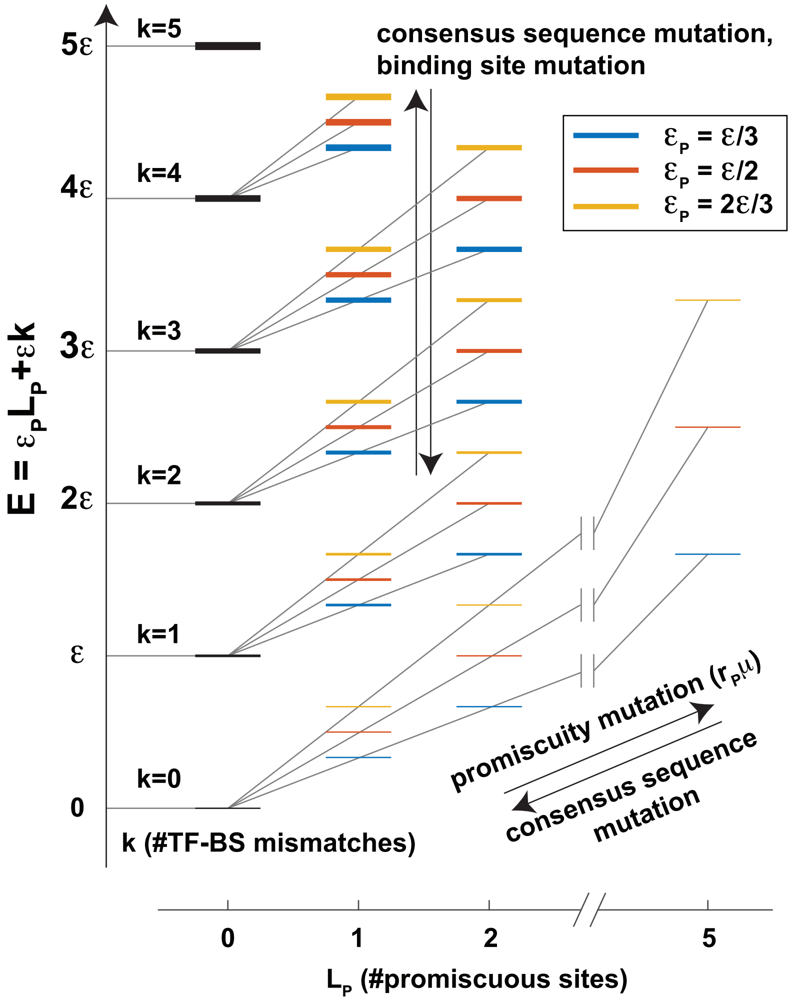

Supplementary Figure 30: **Total TF-DNA binding energies depend on number of mismatches as well as on the number of promiscuous TF positions.** We plot the different energy levels depicting the TF-BS binding energy, $E = \epsilon_P L_P + \epsilon k$, for TFs with varying number of promiscuous positions L_P and k mismatches between the TF and BS in the remaining $L - L_P$ specific positions. Note that lower E corresponds to tighter TF-BS binding. We illustrate this for three different values of ϵ_P, the energy contribution per promiscuous position (different colors). Increasing line thickness of the energy levels represents higher mismatch values k. While promiscuity-promoting mutations increase L_P by converting a specific position to a promiscuous one, regular TF mutations that hit a promiscuous position can convert it to be specific and decrease L_P.

We also introduce an additional type of mutation, called "promiscuity-promoting" mutation, that occurs at rate $r_P\mu$. As illustrated in Fig. 5 of the main text, these mutations convert a specific TF position in the consensus sequence to a promiscuous one. A promiscuous position can return to

be specific again if it is hit by a consensus TF mutation (regular TF mutations we considered until now, happening at rate $r_{TF}\mu$).

Promiscuity entails a cost in terms of TF-BS binding. To elucidate this cost, we consider the dependency of the free (dimensionless) concentration, C_0, of a TF, on the binding preferences of the TF. For a TF with no promiscuous positions, C_0 can be calculated in the chemical potential framework as

$$C_0(L_P = 0) = \frac{C}{GS(\epsilon, L) + \sum_n \exp(-E_n)}, \tag{S17}$$

where C is the copy number of the TF, G is the number of sites on the DNA where the TF can bind in a sequence-specific manner, n enumerates other possible energy configurations of the TF that are sequence-independent (residing in the free solution, or nonspecific binding to DNA), and E_n is the free energy in configuration n. $S(\epsilon, L) = \langle e^{-\epsilon k} \rangle_{P(k)}$ is the similarity between binding sites defined in [11], with $GS(\epsilon, L)$ acting as the Boltzmann factor for all possible specific binding configurations. This term captures the sequestration of TFs on the DNA due to spurious binding. Assuming that the DNA sequence is random, $P(k) \sim B(L, 3/4)$ is the Binomial distribution for the number of mismatches that a random DNA sequence has with a given TF consensus sequence.

For a promiscuous TF with L_P promiscuous positions, we have,

$$\begin{aligned} C_0(L_P) &= \frac{C}{Ge^{-\epsilon_P L_P} S(\epsilon, L - L_P) + \sum_n \exp(-E_n)} \\ &= C_0(L_P = 0) \frac{GS(\epsilon, L) + \sum_n \exp(-E_n)}{Ge^{-\epsilon_P L_P} S(\epsilon, L - L_P) + \sum_n \exp(-E_n)} \\ &= C_0(L_P = 0) \frac{1 + A}{e^{-\epsilon_P L_P} \frac{S(\epsilon, L - L_P)}{S(\epsilon, L_P)} + A}, \end{aligned} \tag{S18}$$

where $A = \frac{\sum_n \exp(-E_n)}{GS(\epsilon, L)}$ is an effective parameter that captures the relative contribution of the Boltzmann factor corresponding to spurious specific binding on the DNA, compared with all other Boltzmann factors. We have assumed that $A = 0.1$ is fixed in our calculations, and the results we present are fairly robust to the value of A. The probability that a binding site is bound by a TF with $L_P > 0$ promiscuous positions and k mismatches with respect to the binding site in the remaining $L - L_P$ positions, assuming no other TF type is present, is

$$p = \frac{C_0(L_P) e^{-\epsilon k - \epsilon_P L_P}}{1 + C_0(L_P) e^{-\epsilon k - \epsilon_P L_P}}. \tag{S19}$$

This probability is plotted in Suppl. Fig. 31 for various k and L_p values. While $C_0(L_P)$ can be greater or lesser than $C_0(L_P = 0)$ depending on the value of ϵ_P, we have $C_0(L_P) e^{-\epsilon_P L_P} < C_0(L_P = 0)$. Hence, as the number of promiscuous positions, L_P, in the TF increases, the binding probability decreases.

For instance, consider a TF with consensus sequence $AAAAA$ (see Suppl. Fig. 31). This TF is specific for A's in all five positions of the binding site sequence. Each mismatch in the binding site

sequence (green positions in the sequences in Suppl. Fig. 31) with respect to $AAAAA$ decreases the binding affinity, and thereby decreases the binding probability. Now consider a promiscuous TF with consensus sequence $A * AAA$, where $*$ denotes a promiscuous position. The second position, independent of the bp in the BS sequence (purple positions in the sequences in Suppl. Fig. 31), decreases the binding affinity, but by a lesser amount than a specific position mismatch (green positions). Hence, the binding probabilities of the promiscuous TF to $AAAAA$, $AGAAA$, $ATAAA$ or $ACAAA$ are equal, and higher than the binding probability of the specific TF to $CAAAA$ or $AGAAA$ or other single-mismatch BS sequences.

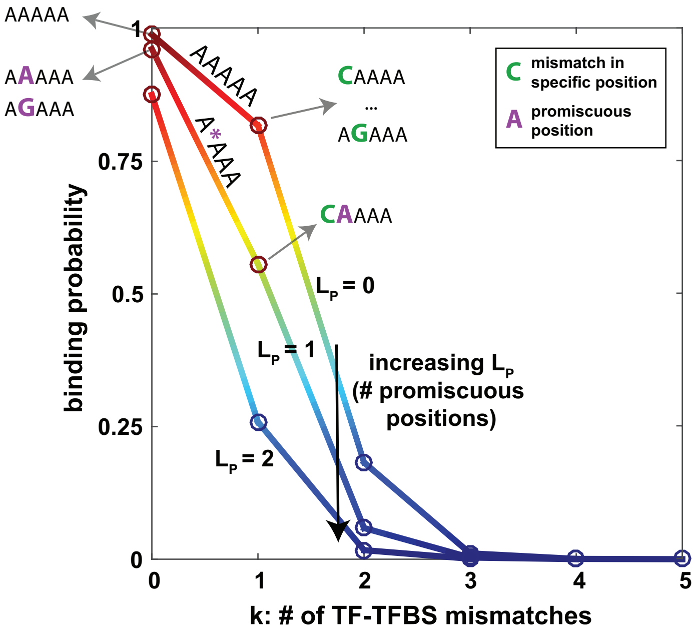

Supplementary Figure 31: **Binding probability of the TF to DNA decreases the more promiscuous it is.** The TF-BS binding probability is plotted as a function of the number of TF-BS mismatches k among the $L - L_P$ specific positions for different values of L_P, the number of promiscuous positions in the TF. We list, as an example, different sequences that are consistent with given (L_P, k).

Steady state after duplication

In the presence of promiscuity-promoting mutations, we obtain the steady state distribution over the genotypic space analytically, from which we obtain the dominant macrostate at steady state for different ρ and Ns values (Suppl. Fig. 32). The inclusion of promiscuity-promoting mutations does not significantly change the dominant macrostate phase plot except for a slight increase in the range of `One TF Lost` macrostate.

We also plot the mean number of promiscuous positions at steady state in Suppl. Fig. 33. This number decreases with selection intensity, because promiscuous positions decrease the TF binding probability (see Suppl. Fig. 31) making them less favorable once specialization has occurred.

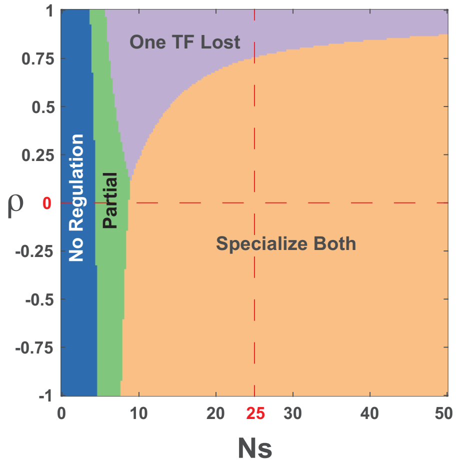

Supplementary Figure 32: **Most probable macrostate in the presense of promiscuity-promoting mutations.** We plot the most probable macrostate at steady state, z^*_{SS}, for different ρ and Ns, for $n_G = 2$ and relative mutation rate $r_P = 3$, keeping other parameters at their baseline values. We choose $r_P = 3$ so that at each position, a specific bp has equal effective mutation rate towards a promiscuous state or another specific bp.

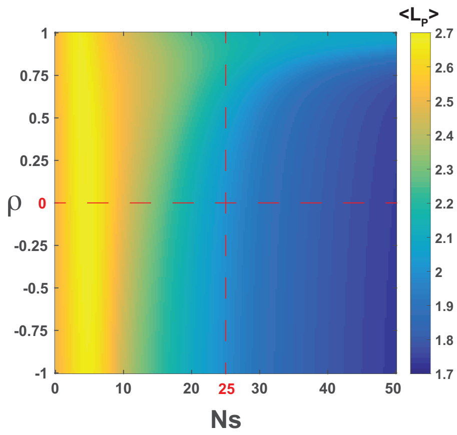

Supplementary Figure 33: **Mean number of promiscuous TF positions at steady state decreases with selection intensity.** We plot the mean number of promiscuous positions at steady state, $\langle L_P \rangle$ (out of $L = 5$), for different values of signal correlation ρ and selection strength Ns. Steady state values of $\langle L_P \rangle$ are within a relatively small range. As selection strength increases, $\langle L_P \rangle$ decreases, yet still remains above zero. Parameter values: $n_G = 2$, $r_P = 3$; other parameters are at their baseline values.

Evolutionary dynamics

Time to specialization

In general, promiscuity-promoting mutations accelerate specialization, as shown in Suppl. Fig. 34. The speedup of the fast `Partial` pathway (3 and 4) is not very large, but the speedup of the slow `Partial` (2 and 5) and the slow `One TF Lost` (1) pathways is considerable, an effect that increases with increasing Ns (see Suppl. Fig. 28 for details of the pathways). Promiscuity-promoting mutations act by converting deleterious BS mutations into neutral or beneficial ones. By that they

effectively lower or even remove fitness barriers. This effect is more significant with a large number of downstream genes, where more constraints on TF evolution exist. The fraction of different pathways does not change much if promiscuity-promoting mutations are present. Note that as a function of Ns, the fraction of fast `Partial` pathways does not change considerably, but the fraction of slow `Partial` pathways decreases while increasing the fraction of slow `One TF Lost` pathways.

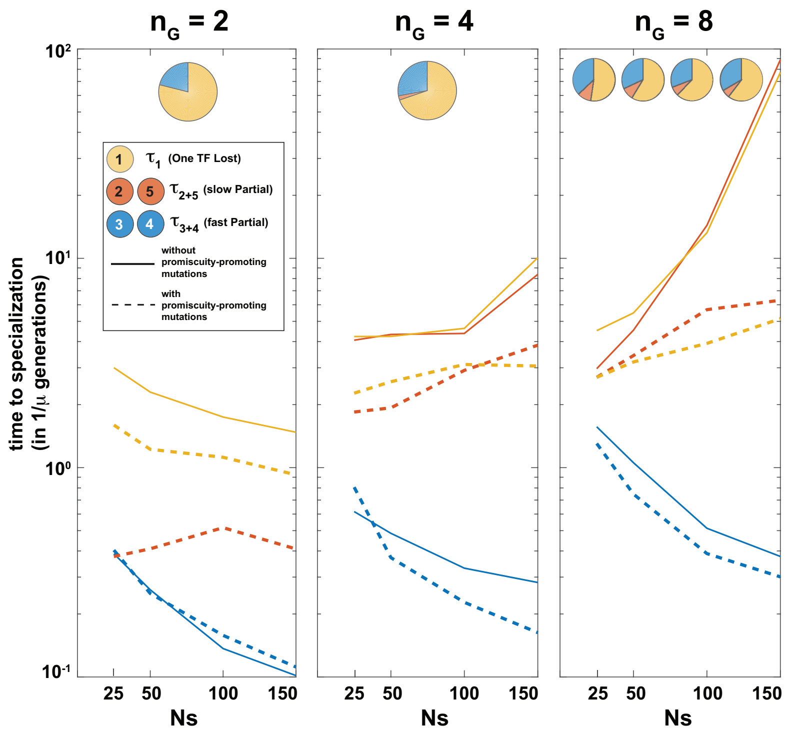

Supplementary Figure 34: **Promiscuity-promoting mutations accelerate specialization.** We plot the times to specialization via different pathways that are depicted in Suppl. Fig. 28, as a function of Ns for different values of n_G (the number of downstream genes per TF), in the absence (solid lines) and presence (dotted lines) of promiscuity-promoting mutations. Specialization times are shown for the slow `One TF Lost` pathway (numbered 1, yellow), the slow `Partial` pathway (numbered 2 and 5, red), and the fast `Partial` pathway (numbered 3 and 4, blue). In general, promiscuity-promoting mutations shorten evolutionary specialization times. This effect is particularly marked for the slow pathways (`One TF Lost` and slow `Partial`) and for large numbers of downstream genes n_G. The pie charts illustrate the fraction of the various pathways at each n_G value. For $n_G = 8$, we plot the pie charts for the different Ns values marked on the x-axis.

Typical trajectory

Promiscuity-promoting mutations play different roles in different phases of the evolutionary trajectory. While after specialization they are less favorable (because they lower binding affinity and potentially destabilize the specialized state), during adaptation they can facilitate fitness valley crossing. In Suppl. Fig. 35, we plot the trajectory of the average number of promiscuous TF positions as a function of time. Starting with no promiscious positions in the `Initial` state, the number of promiscuous positions increases during the transient `One TF Lost` state, and then decreases to reach its steady state value after reaching the `Specialize Both` state. The speedup of evolution is mainly during the transient `One TF Lost` phase, where the number of promiscuous

positions peaks.

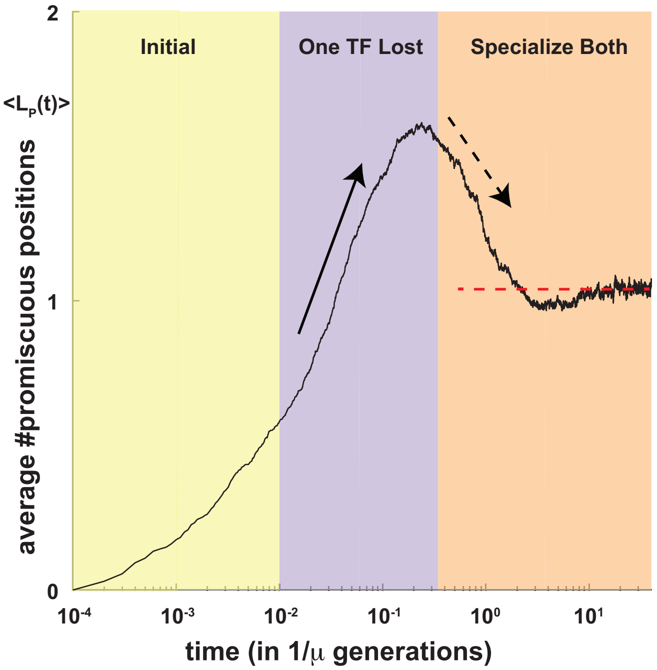

Supplementary Figure 35: **Number of promiscuous positions transiently peaks during adaptation and relaxes after specialization to an intermediate steady state value.** We plot the average number of promiscuous positions $\langle L_P(t) \rangle$ as a function of time for $L = 5, n_G = 4, Ns = 250$ and $r_P = 10$; other parameters are at baseline values. Solid black arrow indicates the increase in the number of promiscuous positions in the transient `One TF Lost` phase, while the dotted black arrow indicates their decrease after specializing. The red dotted line indicates the steady state value of $\langle L_P \rangle$.

Supplementary Note 7 Comparison between biophysically-realistic model and simple models

Gene duplication literature often studies models with a small number of discrete alleles, for example, binary alleles informing whether TF-BS binding occurs. Throughout this work we employ a different approach by including a biophysical description of TF/DNA interactions. Consequently, a large number of different genotypes can often realize each functional architecture (macrostate), capturing naturally the important effects of neutral processes (mutational entropy). Our framework reduces to biallelic models at $L = 1$ and alphabet size $D = 2$ (and multiallelic version with $D = 4$), so we can directly study the relationship between the results for a biophysically realistic fitness landscape and various common simplifications. We refer to these simpler models with $L = 1$ here as the biallelic-like model. The biallelic-like model cannot reproduce some of the results obtained with the biophysically-realistic model of the main text. In particular, certain important macrostates do not exist in the biallelic-like model. We also find an opposite dependence on time to specialization for the different pathways (`One TF Lost` vs. `Partial`). In Suppl. Fig. 36 we plot the dominant macrostate at steady state for two values of D. For $D = 4$ (right panel of the figure), many qualitative features are retained from the more realistic main text model: for instance, the change from `No Regulation` to `Partial` to `Specialize Both` as Ns increases, and the

change from `Specialize Both` to `Specialize Binding` to `One TF Lost` as ρ increases. For $D = 2$, we have `Partial` macrostate dominating at $Ns = 0$, because its entropy is larger than that of the `No Regulation` macrostate. Also, at large Ns and large ρ, `Partial` dominates via the genotypes in which all TF-BS links are strong but the signal sensing domain is not specialized.

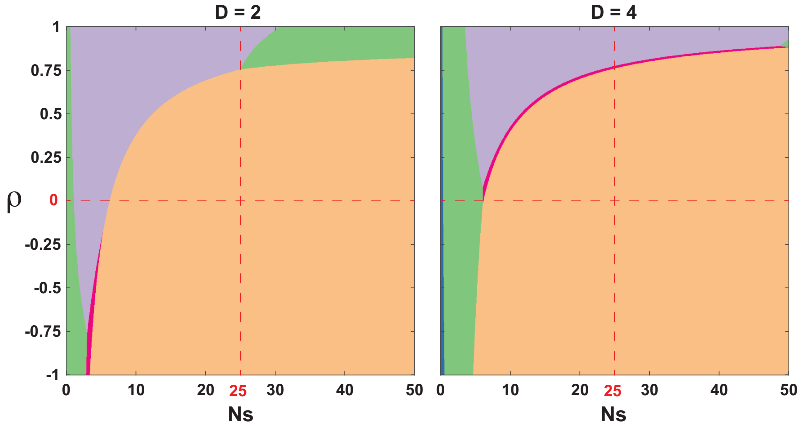

Supplementary Figure 36: **Dominant macrostate at steady state for biallelic-like models.** Here we plot the dominant macrostate at steady state as a function of Ns and ρ for biallelic-like models with alphabet size $D = 2$ (left panel) and $D = 4$ (right panel). Color code used to indicate different macrostates is the same as in the main text.

Certain variants of `Partial` that exist in the general model do not exist in the biallelic-like model, as shown in Suppl. Fig. 37. These states have intermediate fitness and they arise in the fast `Partial` pathway of the main text model, where they form a bridge between the `Initial` and the `Specialize Both` macrostates. Hence, in biallelic models, fast `Partial` pathways do not exist and instead, passing through `Partial` entails either losing a BS or specializing very fast in the signal sensing domain. These states have low fitness in the biallelic-like model and hence `Partial` pathway is actually slow. This is plotted in Suppl. Fig. 38.

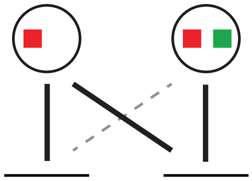

Supplementary Figure 37: **This type of `Partial` macrostate is absent in biallelic-like models.** In biallelic-like models, strong TF-BS link means an exact match between TF and BS. Hence, the description of **`Partial`** states of the kind shown here is impossible.

In summary, biallelic-like models and the biophysically realistic model share a few similarities but also differ in certain important aspects. Biallelic-like models, while being very simplistic, still capture a few key qualitative features of the steady state distribution, for example, the transitions of dominant macrostates along the ρ and Ns axes. On the other hand, biallelic-like models paint a completely different picture of evolutionary dynamics and timescales. Because they do not consider intermediate-fitness `Partial` states, unlike in the biophysically realistic model, time to specialization through `Partial` becomes slower than through `One TF Lost`.

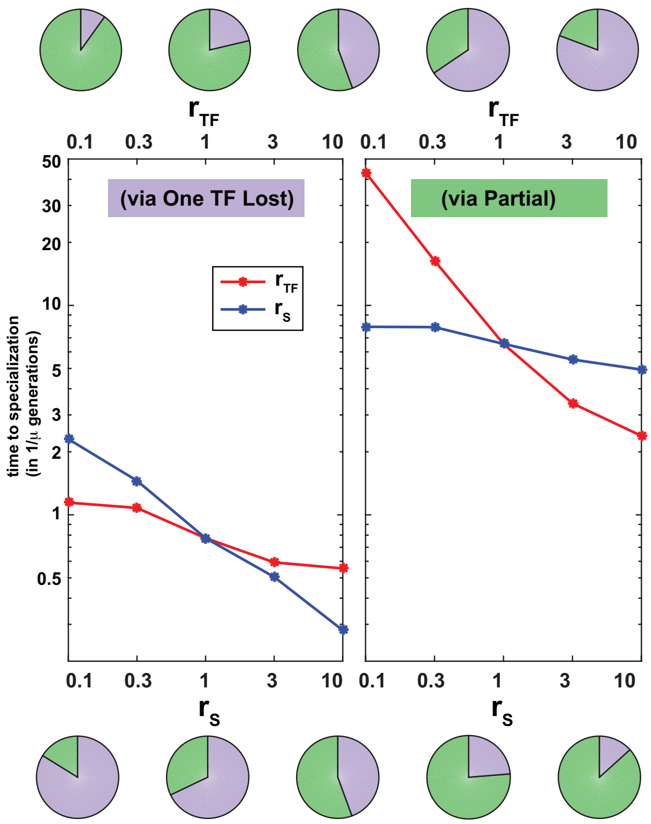

Supplementary Figure 38: **Biallelic-like models reverse the relation between different pathways to specialization: `Partial` pathways are the slow ones and `One TF Lost` pathways are faster, in contrast to the full model studied in the main text.** We plot the times to specialization via `One TF Lost` (left panel) and via `Partial` (right panel), at $Ns = 100$, while changing r_{TF} (red curve) and r_S (blue curve) separately, keeping the other parameters at their baseline values in each case. We also show the fraction of these pathways as pie charts (upper pie charts refer to different r_{TF} values; lower ones to different r_S values).

Supplementary Methods

Markov chain formulation

As explained in Supplementary Note 1, we assume that the time between the emergence and fixation of a beneficial mutation is much shorter than the time until the emergence of the next beneficial mutation. Hence, by neglecting the times between emergence and fixation (or loss) of mutations the population can be captured at any time by a single genotype. This so-called "fixed state assumption" lets us describe the state of the population as a probability distribution over the possible genotypes, $P(\mathcal{D}, t)$ or as a probability distribution over the possible reduced-genotypes, $P(\mathcal{G}, t)$. This can be obtained via a continuous-time discrete-space Markov chain defined over the genotype space $\mathcal{D}$ or the reduced-genotype space $\mathcal{G} = \{M, k_{ij}, \sigma_i\}$. The transition rate between y and x, where either $x, y \in \mathcal{D}$ are genotypes, or $x, y \in \mathcal{G}$ are reduced-genotypes, is the rate of substitution [4]:

$$r_{xy} = 2N\mu_{xy}\Phi_{y\to x} \tag{S20}$$

where N is the population size, μ_{xy} is the mutation rate from (reduced-) genotype y to (reduced-) genotype x, and $\Phi_{y\to x}$ is the probability of fixation of a single copy of x in a population of y (Eq. (S3)). As the probability of fixation $\Phi_{y\to x}$ depends on x and y only via their fitness values $F(x)$ and $F(y)$, and μ_{xy} can be obtained analytically for reduced-genotypes, it is sufficient to consider the Markov chain on the space of reduced-genotypes $\mathcal{G} = \{M, k_{ij}, \sigma_i\}$ rather than on the whole genotype space $\mathcal{D}$. Each reduced-genotype $x = (M, k_{ij}, \sigma_i)$ can be realized by multiple genotypes (DNA sequences), whose number is given by $N_{\text{seq}}(k_{ij}|M)$ (Eq. (S23) and Eq. (S24)) below. Now, the evolution of the probability distribution $P(\mathcal{G}, t)$ is captured by

$$\frac{\partial P(\mathcal{G}, t)}{\partial t} = \mathbf{R}P(\mathcal{G}, t), \tag{S21}$$

where $\mathbf{R}$ is the transition rate matrix of the underlying Markov chain where each entry r_{xy} denotes the rate of transition from y to x.

Steady state after duplication

The probability distribution at steady state, $P_{\text{SS}}(\mathcal{G}) = P(\mathcal{G}, t \to \infty)$, is the non-trivial solution of $\mathbf{R}P_{\text{SS}}(\mathcal{G}) = 0$. It is also possible to obtain $P_{\text{SS}}(\mathcal{G})$ by invoking the set of detailed balance conditions, $r_{xy}P_{\text{SS}}(y) = r_{yx}P_{\text{SS}}(x), \forall x, y$. This results in an elegant expression

$$P_{\text{SS}}(\mathcal{G}) = P_0(\mathcal{G}) \exp(2NF(\mathcal{G})), \tag{S22}$$

where P_0 is the neutral distribution of reduced-genotypes and N is the population size.

To calculate the neutral distribution P_0 of the reduced-genotypes, we begin by enumerating the number of possible BS sequences j that have mismatch values (k_{1j},k_{2j}) with respect to two TFs that match each other at M out of L consensus positions. This number equals:

$$
\begin{aligned}
N_{\text{seq}}(k_1,k_2|M) &= \sum_{j_0=j_0^{\text{min}}}^{j_0^{\text{max}}} \binom{M}{j_0} 3^{M-j_0} \binom{L-M}{L-j_0-k_1} \binom{j_0+k_1-M}{L-j_0-k_2} 2^{k_1+k_2+2j_0-L-M} \\
j_0^{\text{min}} &= \max(\max(0, M-\min(k_1,k_2)), \lceil \frac{L+M-k_1-k_2}{2} \rceil) \\
j_0^{\text{max}} &= \min(M, L-\max(k_1,k_2))
\end{aligned}
\tag{S23}
$$

where for brevity we write k_1, k_2 instead of k_{1j}, k_{2j}, and $\lceil x \rceil$ is the ceiling function, which maps x onto the nearest integer larger than or equal to x. Now, the neutral distribution is (up to proportionality constant)

$$
P_0(x) \sim N_{\text{seq}}(k_{11}, k_{21}|M) N_{\text{seq}}(k_{12}, k_{22}|M) \binom{L}{M} 3^{L-M}. \tag{S24}
$$

From Eq. (S22) we obtain the steady state distribution over the macrostate space. For every macrostate $z \in \mathcal{M}$ the probability to be in this macrostate at steady state equals the sum of probabilities of being in all reduced-genotypes x that are assigned to that macrostate

$$
Q_{\text{SS}}(z) = \sum_{x \in S_z} P_{\text{SS}}(x). \tag{S25}
$$

Free fitness

In thermodynamics and statistical physics the free energy of a system defined as $F = E - TS$, is a state variable which combines energy E and entropy S. At equilibrium, the free energy of a mechanically isolated system kept at equilibrium is minimal. Alternatively, one can use the statistical definition of free energy [12]

$$
F = -\frac{1}{\beta} \log \left(\sum_r g_r e^{-\beta E_r} \right) \tag{S26}
$$

where E_r is the energy of microstate r of the system, g_r is its weight or multiplicity and summation is over all microstates r the system can occupy. Previous work drew an analogy between statistical physics and evolutionary dynamics, where the energy of a microstate E_r was analogous to fitness F of a particular genotype and $\beta = 1/k_B T$ to the population size $2N$. One can follow a similar rationale and define free fitness $\hat{F}$ for each macrostate z [10]:

$$
\hat{F}(z) = \frac{1}{2N} \log \Big[\sum_{x \in S_z} P_0(x) e^{2NF(x)} \Big], \tag{S27}
$$

where summation is over all genotypes x that belong to this macrostate $x \in S_z$ and $P_0(x)$ is the neutral probability of genotype x. The free fitness also equals the likelihood of being at that macrostate at steady state:

$$
\hat{F}(z) = \frac{1}{2N} \log Q_{\text{SS}}(z), \tag{S28}
$$

where $Q_{\text{SS}}(z)$ is analogous to the partition function in statistical physics.

Dominant macrostate

We denote the most probable macrostate at steady state by

$$z^*_{\mathrm{SS}} := \underset{z\in\mathcal{M}}{\arg\max}\; Q_{\mathrm{SS}}(z). \tag{S29}$$

Evolutionary dynamics

We obtain the evolutionary dynamics of $P(\mathcal{G}, t)$ in units of generation time t_g by numerically integrating the Markov chain in time-steps corresponding to one generation:

$$P(\mathcal{G}, t + t_g) = (\mathbf{I} + \mathbf{R}t_g)P(\mathcal{G}, t). \tag{S30}$$

We define $\mathbf{A} = \mathbf{I} + \mathbf{R}t_g$ as the transition probability matrix in this time-unit. From $P(\mathcal{G}, t)$, we obtain the macrostate dynamics (Eq. (S21)) $Q(\mathcal{M}, t)$. For every $z \in \mathcal{M}$,

$$Q(z, t) = \sum_{x\in S_z} P(x, t). \tag{S31}$$

Dominant macrostate

To follow the macrostate dynamics in a more compact way, we refer to the most probable macrostate at each time-point t

$$z^*(t) := \underset{z\in\mathcal{M}}{\arg\max}\; Q(z, t) \tag{S32}$$

as the dominant macrostate at that time.

Time to reach a particular macrostate

We compute the mean first hitting time, $T_{S\leftarrow x}$, to any subset of reduced-genotypes, S, from any other reduced-genotype x, by using the following recursive equation.

$$T_{S\leftarrow x} = t_g + \sum_{y} a_{yx} T_{S\leftarrow y}, \tag{S33}$$

where a_{yx} are elements of the transition probability matrix $\mathbf{A}$. We consider subsets S_z of genotypes that belong to a particular macrostate z, and compute the mean first hitting times, $T_{S_z\leftarrow x}$, to this macrostate. In particular, we compute the mean first hitting times to `Specialize Both`, which we refer to as the "time to specialization", $\tau(x)$.

Dwell times

For every macrostate z, we also compute the dwell time, $t^{dwell}(z)$, which is the mean time to "escape" from that macrostate into any other macrostate z'. For every genotype x in S_z, the mean time to escape from S_z is by definition $T_{S'_z \leftarrow x}$, the mean time taken to hit $S'_z = \mathcal{G} - S_z$, the complementary set of S_z. We define the dwell time in macrostate z as

$$t^{dwell}(z) := \langle T_{S'_z \leftarrow x} \rangle_{x \in S_z} \tag{S34}$$

Stochastic simulations

In addition to analytical solutions of the Markov chain formulation we also used stochastic simulations of TF and BS evolution to validate our analytical solution and also to test additional cases that were not analytically solvable, such as the case where each TF post-duplication regulates multiple genes.

Gillespie Simulation - main model

We use the Gillespie Stochastic Simulation Algorithm [13] to track the evolutionary trajectories of the system. Since we employ the fixed-state assumption, the time to fixation of each mutation is small compared to the waiting time between mutations and we neglect it in the calculations. At each simulation run we obtain a temporal series, $s_0, s_1, s_2, \ldots$, of genotypes (DNA sequences of TF consensus sequence and binding sites, along with signal sensing alleles), and a corresponding sequence of times, $t_0 = 0, t_1, t_2, \ldots$, at which substitutions between consecutive genotypes occurred. Here, s_0 is the initial DNA sequence with which we start the simulation. We construct s_0 by sampling a genotype from the steady state before duplication (with only 1 TF). For every i, from t_i to t_{i+1}, the DNA sequence of the system is s_i, from which there is a substitution event to s_{i+1} at t_{i+1}. We obtain s_{i+1} by appropriately sampling substitutions available from s_i, which can occur via TF consensus sequence mutations, or TF sensing domain mutations, or BS sequence mutations. We also draw $t_{i+1} - t_i$ (the waiting time) from the appropriate exponential distribution in the Gillespie framework. For each DNA sequence s_i, one can obtain the reduced-representation (M, k_{ij}, σ_i). From this, we obtain, for each simulation run r, the time trajectories of reduced-genotypes, $x_r(t)$, starting from $x_r(t = 0) = x_{r0}$. By running multiple times and computing the fractions of runs with each reduced-genotype x at each t, we obtain the dynamical trajectory of the probability distribution of reduced-genotypes, $P^{sim}(\mathcal{G}, t)$, and the steady state distribution, $P^{sim}_{\mathrm{SS}}(\mathcal{G})$. Grouping the reduced-genotypes into macrostates, we also obtain the dynamical trajectory of the probability distribution of macrostates, $Q^{sim}(\mathcal{M}, t)$ and steady state distribution of macrostates, $Q^{sim}_{\mathrm{SS}}(\mathcal{M})$.

The simulations enable us to compute non-trivial *path-dependent* quantities relating to an ensemble of trajectories $\{x_r(t)\}$, as well as to provide full distributions of quantities of interest. One such example is the mean hitting time to some macrostate z, conditioned on not hitting some other particular macrostate on the way. While it is possible in principle to compute such a path-dependent quantity exactly, in practice this requires too much numerical effort and Gillespie simulation becomes the method of choice.

Time to specialization, dependent on pathway

As explained in the main text, for a single trajectory (population), there are two main paths from `Initial` to `Specialize Both`, each with a different dominant "transient state". One pathway is fast and predominantly goes via genotypes in `Partial` macrostate, and the other is slow and predominantly via genotypes in `One TF Lost` macrostate. In each simulation run r, we calculate the time to specialization, and also record the dominant transient state. By running many simulations, we have a set of times to specialization that go via the fast pathway of `Partial` $\{\tau_{fast}\}$, and those via the slow pathway of `One TF Lost` $\{\tau_{slow}\}$. Using these, we obtain the empirical distributions of τ_{slow} and τ_{fast}, their means ($\bar{\tau}_{slow} = \langle\tau_{slow}\rangle$ and $\bar{\tau}_{fast} = \langle\tau_{fast}\rangle$); we also record the fraction of pathways proceeding via the slow and fast alternatives.

Alternative model - fixed signal sensing domain

In the second model variant, as mentioned in Supplementary Note 1, TFs are not equipped with an evolvable signal sensing domain σ_i. The active concentrations of the TFs, $C_i(m)$, in different environments m, are explicitly defined separately. In the stochastic simulation of this model variant, we therefore considered mutations only in the TF consensus sequence and the BS sequences. We also assumed a timescale separation, such that the TF consensus sequences evolve on a slower timescale compared to the BS sequences. We implement this by performing alternating rounds of one TF consensus sequence mutation and 50 BS sequence mutations, resulting in $r_{TF} = 0.02$. These two rounds together are considered a single time step of the simulation, which amounts to counting the number of TF consensus sequence mutations that have arisen.

As in the Gillespie simulation, we choose the starting point by sampling from the steady state before duplication with only 1 TF. The duplicate TF has the same binding preferences as the original TF but has different expression pattern $C_2(m)$ than the first $C_1(m)$. In each round, we calculate the fixation probability of the mutant using Eq. (S3), and compare a randomly drawn number between 0 and 1 to either fix them or not.

In Suppl. Fig. 39 we compare between this stochastic simulation and the analytical solution for the steady state distributions of various mismatches the steady state distribution of the match between the two TFs.

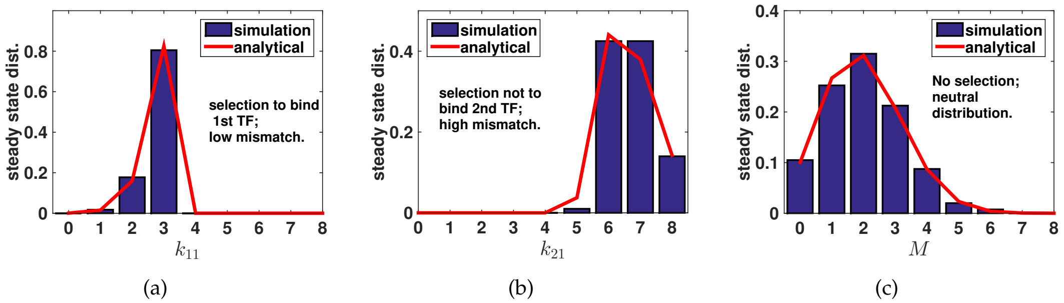

Supplementary Figure 39: **Comparison between stochastic simulation and exact results.** Blue bars represent the statistics over 400 independent runs of the stochastic simulation. Red curve represents the analytical solution for the steady state distribution. We illustrate distributions of k_{11} mismatch between first TF and first gene (a) where selection for the regulation of this gene incurs low mismatch; mismatch between second gene and TF k_{21} where selection here results in high mismatch, such that this gene is NOT regulated by this TF. (c) illustrates the match distribution M between the two TFs in the absence of selection, so that the Bernoulli distribution is obtained. Parameters: $L = 8$, $\epsilon = 3$, $C_0 = 3.269 \times 10^5$.

Supplementary References

[1] Madeline A. Shea and Gary K. Ackers. The OR control system of bacteriophage lambda: A physical-chemical model for gene regulation. *Journal of Molecular Biology*, 181(2):211–230, January 1985.

[2] Ulrich Gerland, J. David Moroz, and Terence Hwa. Physical constraints and functional characteristics of transcription factor-dna interaction. *Proceedings of the National Academy of Sciences*, 99(19):12015–12020, 2002.

[3] P. H. Von Hippel and O. G. Berg. On the specificity of DNA-protein interactions. *Proceedings of the National Academy of Sciences*, 83(6):1608, 1986.

[4] Michael Lässig. From biophysics to evolutionary genetics: statistical aspects of gene regulation. *BMC Bioinformatics*, 8(6):1–21, 2007.

[5] Sebastian J. Maerkl and Stephen R. Quake. A systems approach to measuring the binding energy landscapes of transcription factors. *Science*, 315(5809):233–237, January 2007.

[6] Henrik Kaessmann, Nicolas Vinckenbosch, and Manyuan Long. RNA-based gene duplication: mechanistic and evolutionary insights. *Nature Reviews Genetics*, 10(1):19–31, January 2009.

[7] Nicolas Vinckenbosch, Isabelle Dupanloup, and Henrik Kaessmann. Evolutionary fate of retroposed gene copies in the human genome. *Proceedings of the National Academy of Sciences of the United States of America*, 103(9):3220–3225, February 2006.

[8] Michael M. Desai and Daniel S. Fisher. Beneficial mutation-selection balance and the effect of linkage on positive selection. *Genetics*, 176(3):1759–1798, July 2007.

[9] John H. Gillespie. *Population genetics: a concise guide*. The Johns Hopkins University Press, 2nd edition, July 2004.

[10] Guy Sella and Aaron E. Hirsh. The application of statistical physics to evolutionary biology. *Proceedings of the National Academy of Sciences of the United States of America*, 102(27):9541–9546, July 2005.

[11] Tamar Friedlander, Roshan Prizak, Călin C. Guet, Nicholas H. Barton, and Gašper Tkačik. Intrinsic limits to gene regulation by global crosstalk. *Nature Communications*, 7:12307, August 2016.

[12] R. K. Pathria. *Statistical Mechanics by R. K. Pathria*. Butterworth-Heinemann, 1st edition, 1988.

[13] Daniel T Gillespie. A general method for numerically simulating the stochastic time evolution of coupled chemical reactions. *Journal of Computational Physics*, 22(4):403–434, December 1976.
